# Machine learning identifies prognosticators of intracranial metastatic disease in patients with breast or lung cancer

**DOI:** 10.1038/s43856-026-01609-3

**Published:** 2026-04-23

**Authors:** Marco V. Istasy, Amol Verma, Katarzyna J. Jerzak, Sunit Das

**Affiliations:** 1 Institute for Medical Sciences, University of Toronto, Toronto, ON Canada; 2 Temerty Faculty of Medicine, University of Toronto, Toronto, ON Canada; 3 Institute for Clinical Evaluative Sciences, Toronto, ON Canada; 4 Institute for Health Policy, Management and Evaluation, University of Toronto, Toronto, ON Canada; 5 Department of Medicine, University of Toronto, Toronto, ON Canada; 6 Li Ka Shing Knowledge Institute, St. Michael’s Hospital, Toronto, ON Canada; 7 Department of Laboratory Medicine and Pathology, University of Toronto, Toronto, ON Canada; 8 Division of Medical Oncology, Sunnybrook Odette Cancer Center, Toronto, ON Canada; 9 Division of Neurosurgery, St. Michael’s Hospital, Toronto, ON Canada

**Keywords:** Breast cancer, Lung cancer, Metastasis, CNS cancer, Predictive markers

## Abstract

**Background:**

Intracranial metastatic disease is a severe complication of cancer that confers substantial morbidity and mortality. Patients with breast or lung cancer are at particularly elevated risk of IMD. Early identification of individuals at increased risk could enable targeted surveillance and timely intervention.

**Methods:**

We developed interpretable machine-learning competing-risk models to estimate the risk of intracranial metastatic disease among patients with breast or lung cancer. For each cancer type, cause-specific Cox models were combined via the Aalen–Johansen estimator to produce absolute risk estimates at one, three, and five years.

**Results:**

Here we show high test set discrimination for intracranial metastatic disease (Uno’s C-index: breast 0.95; lung 0.88) and favorable time-dependent precision–recall performance (AUPRC(t) at 1/3/5 years: breast 0.17/0.53/0.63; lung 0.37/0.61/0.64). Decision-curve analysis across relevant thresholds demonstrates greater net clinical benefit than baseline strategies. Model interpretability analysis identifies cancer stage as the dominant determinant in both cancers; in breast cancer, triple-negative and HER2-positive subtypes contribute additional risk, whereas in lung cancer, histology and tumor size are prominent contributors.

**Conclusions:**

Machine-learning based competing-risk survival models offer greater insight into prognostication of intracranial metastatic disease than baseline strategies. These findings support the potential of such models to strengthen personalized risk stratification and guide targeted surveillance for Intracranial metastatic disease among patients with breast or lung cancer.

## Plain language summary

Patients with breast or lung cancer can develop cancer that spreads to the brain, called metastasis, which causes serious illness and shortens life. This study aimed to identify which patients are most likely to develop brain metastasis. The researchers built computer programs using patient information to estimate the chance of developing brain metastasis within one, three, and five years. The programs also accounted for the fact that some patients may die before brain spread occurs. The programs predicted risk well and performed better than simpler statistical approaches. Cancer stage was the strongest predictor in both cancers. These tools could support more personalized follow-up and earlier detection, but they should be tested further in real-world care.

## Abstract (web summary)

Istasy et al. develop interpretable machine learning models to estimate 1-, 3- and 5-year intracranial metastasis risk in breast and lung cancer, accounting for competing mortality. The models demonstrate strong discrimination and net clinical benefit over standard methods, identifying key predictors to guide targeted surveillance.

## Introduction

Intracranial metastatic disease (IMD) occurs in ~30% of patients with metastatic cancer^1–10^. Its incidence is expected to rise due to population aging and advances in systemic cancer therapies that prolong survival^7,8,10^. The likelihood of IMD is strongly influenced by the histological and molecular characteristics of the primary tumor, with certain malignancies exhibiting a greater propensity for central nervous system dissemination. Notably, lung and breast cancers are the predominant sources of brain metastases, accounting for roughly 60% and 11% of all IMD cases, respectively^2,7,11–16^.

IMD substantially impacts both patient survival and quality of life (QoL)^17–20^. Without intervention, median survival following IMD diagnosis is less than two months^21,22^, whereas current therapeutic modalities, including surgical resection, radiotherapy, and systemic therapy, typically extend survival to less than one year^16,21,23–30^. The complexity of treatment is further compounded by delays in identification: patients with systemic cancer rarely undergo brain imaging in the absence of neurological symptoms, and over 80% present with multiple brain metastases at detection^31^. This delay is clinically significant as interventions that can achieve durable intracranial disease control and improve QoL are most effective when lesions are identified at a small size and in limited numbers^3,32,33^. Nevertheless, routine surveillance imaging of all patients with systemic cancer is neither clinically feasible nor economically sensible^34^. Consequently, there is a pressing need to identify reliable prognostic factors to guide targeted surveillance and to enable earlier detection in patients at elevated risk.

Several risk factors for IMD have been reported in breast and lung cancer populations. These include demographic variables such as younger age at diagnosis^35–38^, general tumor-related characteristics such as higher grade and advanced stage^36,39–42^, and disease-specific factors. In breast cancer, ER-negative, HER2-positive, and triple-negative subtypes^40,43,44^, as well as BRCA1/2 mutations^45,46^, are associated with an increased likelihood of brain metastases. In lung cancer, sex^41,47^, histological subtype^37,47^, and oncogenic drivers such as EGFR, ROS1, and ALK alterations^41^ have been strongly linked to IMD risk.

Despite these associations, no series of predictors has been consistently validated for accurate IMD risk stratification. Indeed, traditional statistical methods have proven inadequate in offering robust predictive algorithms that incorporate both patient- and tumor-specific features^1,40,41,48–50^. Consequently, there is a critical need for more sophisticated predictive models to identify patients at elevated risk of developing IMD.

Machine Learning (ML) offers a promising avenue, with the potential to improve predictive performance beyond that of conventional models. Yet successful integration of ML models into clinical practice requires not only predictive accuracy but also model interpretability. Indeed, clinicians have been found to require a clear understanding of the underlying factors driving a model’s predictions to confidently incorporate such tools into patient care^51,52^.

In this study, we hypothesize that interpretable ML approaches can significantly improve the prediction of IMD in patients with breast or lung cancer. We present a comprehensive and cancer-agnostic ML paradigm with excellent discriminative ability for identifying high-risk individuals. Furthermore, we interrogate the decision-making process of the algorithms to elucidate clinically relevant predictors of metastatic spread.

In this study, we develop interpretable machine-learning models to estimate individual risk of intracranial metastatic disease among patients with breast or lung cancer while accounting for the competing risk of death. The models show strong discrimination and favorable precision–recall performance in held-out testing, and decision analysis shows greater net clinical benefit than baseline strategies across clinically relevant risk thresholds. Model-interpretation analyses identify disease stage as the dominant driver of risk in both cancers, with additional contributions from breast cancer subtype, including triple-negative and HER2–positive disease, and, in lung cancer, histology and tumor size. Together, these findings support the use of interpretable machine-learning risk models to improve personalized risk stratification and to inform targeted surveillance for earlier detection of IMD.

## Methods

### Study population and variables

This study utilized a population-based dataset obtained from the Ontario Cancer Registry, which is the provincial database of information about all Ontario residents diagnosed with cancer. The registry is housed at ICES, formerly known as the Institute for Clinical Evaluative Sciences, an independent, non-profit research institute whose legal status under Ontario’s health information privacy law allows it to collect and analyze health care and demographic data, without consent, for health system evaluation and improvement.

The dataset utilized in this study included all patients from the Canadian province of Ontario from January 1, 2010 to August 30, 2023 with a diagnosis of breast cancer (ICD-10 C50.0–C50.9) or lung cancer (ICD-10 C34.0–C34.9). Exclusion criteria were: (1) age <18 or >105 years at diagnosis, (2) more than one primary cancer diagnosis, and (3) prior diagnosis of intracranial metastatic disease (IMD). Time zero was defined as the date of primary cancer diagnosis.

IMD was ascertained from three provincial databases: Cancer Activity Level Reporting, Discharge Abstract Database, and the National Ambulatory Care Reporting System using pathology reports, radiology reports, treatment codes, and hospitalization records using ICD-10-CA and ICD-O-3 codes. A prespecified ICES code list was applied uniformly from Jan 1, 2010 to Aug 30, 2023; ICD-10-CA (v2018, v2022) and ICD-O-3 (v2013, v2019) version updates did not affect our definitions. Patients were considered IMD-positive if any of the three databases recorded IMD, with the first coded instance serving as the diagnosis date. Synchronous IMD, defined as ≤1 week from initial cancer diagnosis, was excluded, whereas only metachronous IMD, defined as >1 week from diagnosis, was included. Follow-up continued until death, loss to follow-up, or end of data availability (August 2023). Loss to follow-up was defined as no provincial health system encounter for >1 year or expiration of the provincial health card without renewal for >1 year.

We had the opportunity to assess the following variables in both cohorts: age at diagnosis, tumor size, tumor site (according to the ICD-10 topography codes), histology (according to the ICD-O-3 morphology codes), laterality, and stage. Information regarding hormone receptor, HER2, and triple-negative status and tumor grade were also included in the breast cancer cohort, while sex was included in the lung cancer cohort. Features were selected to balance clinical relevance and practical accessibility, prioritizing variables commonly available in standard oncology datasets. The full feature space is represented in Supplementary Tables S10, S11.

To characterize patient comorbidities, we employed the Johns Hopkins ACG® System. Similar to previous work^53^, all diagnoses associated with hospital admissions and physician billing claims within two years prior to primary cancer diagnosis were identified for each patient. These diagnoses were then mapped to their corresponding ICD codes and categorized into 31 distinct Aggregate Diagnosis Groups (ADGs; see Supplementary Table S12). For each patient, an aggregate ADG score was then calculated as previously described^53^.

The dataset was linked using unique encoded identifiers and analyzed at ICES.

### Data preprocessing

The dataset was randomly partitioned into a development set (70%) and a held-out test set (30%). Stratified sampling was applied to ensure proportional representation of patients with and without IMD across both sets. All model training and hyperparameter optimization were conducted exclusively within the development set. Within this development set, a five-fold cross-validation strategy was employed: in each fold, 80% of the data were used for training and 20% for validation. This approach provided robust internal performance estimates while mitigating overfitting. The held-out test set remained untouched during model development and was used only for final performance evaluation.

Missing values were imputed separately within the development and test sets to prevent data leakage. For numeric variables, imputation was performed using the mean value from the training portion of each fold; for categorical variables, the mode was used. The imputation statistics derived from training data were consistently applied to the corresponding validation and test subsets. Fewer than 20% of entries required imputation per feature (Supplementary Table S1). Mean and mode imputation was selected to minimize bias and ensure reproducibility, given the modest extent of missingness and the absence of features with extensive missingness. Outliers were retained to preserve the full spectrum of clinical and demographic variability.

For categorical predictors, categories with prevalence <5% in the development set were collapsed into an ‘Other’ level to avoid sparse strata.

### Statistical analysis

For each cancer type, we fit two cause-specific Cox proportional hazards models, one for IMD and one for death without prior IMD, using a subset of the above covariates. Estimation used the Efron method for ties and robust standard errors; no clustering or stratification terms were included. Right-censoring for time-dependent evaluation was handled using inverse probability of censoring weights (IPCW; see Model Evaluation). All analyses were conducted in Python 3.9 on a Linux-based ICES high-performance computing cluster.

### Predictive modeling

We implemented cause-specific machine-learning Cox models using the CatBoost framework^54^. For each cancer cohort, two models were trained: one to estimate the cause-specific hazard for IMD and one for death without prior IMD. Model development and hyperparameter tuning were conducted exclusively within the development set (70%), leaving a held-out test set (30%) untouched for final evaluation. Within the development set, we used five-fold cross-validation, with folds created via stratified sampling by IMD and death occurrence. In each fold, the training partition was used for fitting and the validation partition for hyperparameter selection. The optimization criterion was Uno’s C-index computed on the validation data. After tuning, the final model for each endpoint was refit on the full development set with the selected hyperparameters and evaluated on the test set.

Hyperparameters were optimized with Optuna^55^ using broad, default-centered search ranges; early stopping was not used. No standardization, scaling, or one-hot encoding was required, as CatBoost natively handles heterogeneous feature types. No oversampling or class-imbalance procedures were applied. Categorical and continuous predictors were as aforementioned; continuous variables were modeled linearly, and no interaction terms were included. Patients contributed at most one outcome per endpoint: IMD events were treated as events for the IMD model and as censoring for the death model, while deaths without prior IMD were treated as events for the death model and as competing events for the IMD process.

To obtain patient-level cumulative incidence predictions under competing risks, the two fitted cause-specific hazard functions (IMD; death) were combined via the Aalen–Johansen estimator, yielding cumulative incidence functions (CIFs) for IMD at clinically relevant horizons. All modeling steps were performed separately for breast and lung cancer, producing two disease-specific CIF models, each derived from its corresponding pair of cause-specific CatBoost Cox models.

### Model evaluation

After hyperparameters were selected, each cause-specific model (IMD; death without prior IMD) was refit on the full development set and assessed on the independent 30% test set. For the cause-specific hazard models, discrimination was quantified by Uno’s C-index.

For all time-dependent metrics, the cumulative/dynamic case–control definition was employed: at horizon ***t***, cases were defined as patients who developed IMD by ***t*** while controls as those event-free by ***t****;* death before ***t*** was treated as a competing event with such patients exiting the risk set at their death time. Right-censoring was addressed with inverse probability of censoring weighting (IPCW) by inverting the censoring survival function estimated non-parametrically using the Kaplan–Meier estimator. The IPCW scheme was then stabilized by truncating weights at the 1st and 99th percentiles to limit the influence of extreme estimates prior to renormalized at each evaluation time. All IPCW quantities were recomputed within each CV fold and were re-estimated on the held-out test set to avoid leakage and reflect split-specific censoring.

For the Aalen–Johansen–derived cumulative incidence functions (AJ-CIFs), time-dependent predictive accuracy at 1, 3, and 5 years was summarized using IPCW-based AUROC(t) and AUPRC(t) estimators implemented in custom code, and overall predictive accuracy was measured by the Integrated Brier Score (IBS) integrated over 0–5 years, with absolute and relative error reduction computed against a null model. Calibration was evaluated using calibration-in-the-large at 1, 3, and 5 years and the Integrated Calibration Index summarized over time, both computed with IPCW and smoothed calibration curves. Uncertainty for AUROC(t), AUPRC(t), IBS, calibration metrics, and CIF estimates was obtained via 5000 pairs-bootstrap resamples of the test set to form 95% confidence intervals; cross-validation results are presented as mean ± SD without additional inferential testing.

Clinical utility was assessed using time-dependent decision-curve analysis at 1, 3, and 5 years, using each model’s predicted IMD cumulative incidence (AJ-CIF) at the corresponding horizon. For each threshold probability (evaluated from 0% to 10% in 0.5 percentage-point increments), patients with predicted IMD risk at or above the threshold were considered test-positive (i.e., selected for surveillance/intervention). We computed net benefit on the held-out test set by balancing the proportion of correctly selected patients who developed IMD by the horizon against the proportion of selected patients who did not, with false positives penalized according to the chosen threshold. For interpretability, curves were compared to the standard treat-none and treat-all reference strategies. Right-censoring was handled using IPCW consistent with the time-dependent performance metrics, with death prior to the horizon treated as a competing event. Uncertainty bands were obtained via bootstrap resampling. The 0–10% threshold range was chosen a priori to focus on clinically plausible action thresholds for IMD surveillance^1,2^.

For context, we trained two comparator models under the same pipeline: a minimal age-and-stage-only tree-ensemble baseline and a standard multivariable linear Cox model using the entire covariate set.

### Feature importance

Feature importance was evaluated for each cause-specific CatBoost Cox model using two complementary approaches. First, global Shapley Additive exPlanations (SHAP) importance was summarized as the mean absolute SHAP value per feature, computed out-of-fold and aggregated for reporting on the held-out test set. Second, permutation-based importance was quantified as the decrement in Uno’s C-index induced by feature-wise shuffling. To synthesize these perspectives, we formed a consensus ranking using the rank-product of the two method-specific ranks, breaking ties by average rank. Consensus ranks and the constituent SHAP and permutation orders are reported per endpoint (IMD; death) and per cancer (breast; lung). Directionality of associations was interpreted from the cause-specific Cox hazard ratios rather than from SHAP or permutation metrics.

### Ethical consideration

ICES is a prescribed entity under Ontario’s Personal Health Information Protection Act (PHIPA). Section 45 of PHIPA authorizes ICES to collect personal health information, without consent, for the purpose of analysis or compiling statistical information with respect to the management of, evaluation or monitoring of, the allocation of resources to or planning for all or part of the health system. Projects that use data collected by ICES under section 45 of PHIPA, and use no other data, are exempt from REB review. The use of the data in this project is authorized under section 45 and approved by ICES’ Privacy and Legal Office. All analyses were performed on de-identified data in a secure environment, and no individual-level results are reported.

## Results

### Patient cohorts and time-to-IMD

Using the Ontario Cancer Registry, we identified 86,082 patients with breast cancer and 57,259 with lung cancer diagnosed between 2010 and 2023. Median follow-up by the reverse Kaplan–Meier method was 7.84 years (95% CI, 7.81–7.88; IQR, 5.24–10.55) in the breast cancer cohort and 7.36 years (95% CI, 7.27–7.44; IQR, 4.85–10.05) in the lung cancer cohort. During follow-up, 2,665 (3.1%) breast cancer patients and 6,426 (11.2%) lung cancer patients developed IMD, while 15,865 (18.4%) and 39,065 (68.2%) died without prior IMD, respectively; 67,552 (78.5%) in the breast cohort and 11,768 (20.6%) in the lung cohort were censored without IMD or death (Table 1).

**Table 1:** Cohort characteristics and outcomes for breast and lung cancer patients

	Breast cancer	Lung cancer
Cohort size, *n*	86,082	57,259
IMD events, *n* (%)	2665 (3.1%)	6426 (11.2%)
Death without prior IMD, *n* (%)	15,865 (18.4%)	39,065 (68.2%)
Censored, *n* (%)	67,552 (78.5%)	11,768 (20.6%)
Follow-up, years — median (95% CI)	7.84 (7.81–7.88)	7.36 (7.27–7.44)
Time to IMD among cases, years — median (95% CI)	2.49 (2.40–2.60)	0.79 (0.77–0.82)
Time to death without IMD, years — median (95% CI)	3.58 (3.51–3.64)	0.57 (0.56–0.59)
IMD incidence rate, per 100 PY (95% CI)	0.45 (0.43–0.47)	4.54 (4.43–4.65)
Death incidence rate, per 100 PY (95% CI)	2.68 (2.64–2.73)	27.61 (27.34–27.88)

Follow-up by reverse Kaplan–Meier. Incidence rates are per 100 person-years.

*IMD* intracranial metastatic disease, *CIF* cumulative incidence function, *IQR* interquartile range, *PY* person-years.

**Fig. 1:**
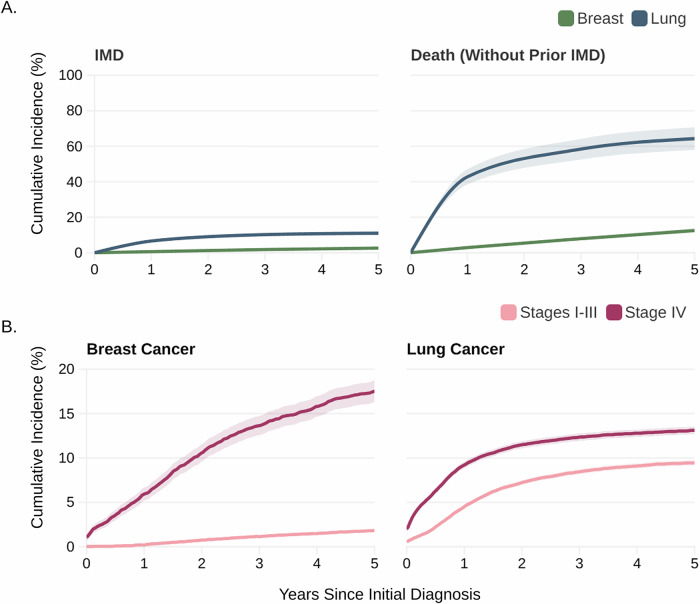
Cumulative incidence under competing risks. **A** Cumulative incidence functions (CIFs) IMD and for death without prior IMD in breast (green; *n* = 86,082) and lung (blue; *n* = 57,259) cancer over 5 years. IMD rates were 0.6%, 1.8%, 2.6% and 6.6%, 10.2%, 11.0% at 1, 3, 5 years for breast and lung cancer, respectively. Death without IMD rates were 2.9%, 7.9%, 12.5% and 42.8%, 58.4%, 64.3%. for breast and lung cancer, respectively. Both the IMD CIF and the death-without-prior-IMD CIF differed significantly between breast and lung cancer over 0–5 years (Gray’s test, *p* < 0.001). Shaded bands denote 95% CIs. **B** Stage-stratified CIFs of IMD under competing risk of death. In breast cancer, Stage IV carried higher absolute risk than Stages I–III by 5.5, 12.3, and 15.5 percentage points at 1, 3, and 5 years, respectively; in lung cancer, the corresponding differences were 4.6, 3.8, and 3.6 percentage points. Stage-stratified CIFs differed significantly by Gray’s test (*p* < 0.001).

Cumulative incidence functions (CIFs) with death treated as a competing risk differed markedly by cancer type (Fig. 1A). In breast cancer, IMD CIFs were 0.6%, 1.8%, and 2.6% at 1, 3, and 5 years, respectively; in lung cancer, the corresponding CIFs were 6.6%, 10.2%, and 11.0%. CIFs for death without prior IMD were 2.9%, 7.9%, and 12.5% in breast cancer versus 42.8%, 58.4%, and 64.3% in lung cancer (95% CIs in Supplementary Table S1). Differences over 0–5 years were significant by Gray’s test (*p* < 0.001), with absolute separations at 1, 3, and 5 years of 6.0, 8.4, and 8.4 percentage points for IMD and 39.9, 50.5, and 51.8 percentage points for death without IMD, respectively.

Among patients who developed IMD, median time to IMD was 2.49 years (IQR, 1.30–4.37) in the breast cohort and 0.79 years (IQR, 0.26–1.62) in the lung cohort. Incidence rates were 0.45 versus 4.54 IMD events and 2.68 versus 27.61 deaths without prior IMD per 100 person-years in breast and lung cancer, respectively (Supplementary Table S1). Among those who died without prior IMD, median time to death from diagnosis was 3.58 years (IQR, 1.66–6.34) in breast cancer and 0.58 years (IQR, 0.16–1.74) in lung cancer.

### Determinants of IMD under competing mortality

Stage-stratified CIFs were estimated separately for breast and lung cancer (Fig. 1B). In breast cancer, Stage IV carried a higher absolute risk of IMD than Stages I–III by 5.5, 12.3, and 15.5 percentage points at 1, 3, and 5 years, respectively; the corresponding differences in lung cancer were 4.6, 3.8, and 3.6 percentage points.

Consistent with these absolute-risk patterns, cause-specific Cox models for IMD demonstrated graded stage effects. In breast cancer, Stage II was similar to Stage I (hazard ratio [HR] 0.98, 95% CI 0.94–1.02), whereas hazards increased at Stages III–IV (Stage III HR 1.59, 95% CI 1.50–1.68; Stage IV HR 3.64, 95% CI 3.35–3.95). Compared with HR–negative disease, HR-positive status was associated with a lower hazard (HR 0.75, 95% CI 0.72–0.79), whereas HER2-positive (HR 1.19, 95% CI 1.12–1.25) and triple-negative subtypes (HR 1.34, 95% CI 1.25–1.43) were associated with higher hazards. In lung cancer, stage effects were likewise pronounced (Stage III HR 1.53, 95% CI 1.46–1.60; Stage IV HR 2.07, 95% CI 1.99–2.15, both vs Stage I) with differences emerging among histology: squamous cell carcinoma showed a lower IMD hazard (HR 0.73, 95% CI 0.70–0.77) relative to ‘Other’ histologies.

For death without prior IMD, effect sizes were larger and stage remained the dominant determinant. In breast cancer, hazards increased steeply from Stage II (HR 1.21, 95% CI 1.17–1.24) to Stage III (HR 2.06, 95% CI 1.99–2.14) and Stage IV (HR 6.71, 95% CI 6.40–7.03), compared with Stage I. HR-positive (HR 0.79, 95% CI 0.76–0.82) and HER2-positive (HR 0.91, 95% CI 0.87–0.94) status were associated with lower hazard, whereas triple-negative disease increased hazard (HR 1.15, 95% CI 1.10–1.21). In lung cancer, stage was again the principal driver (Stage II HR 1.10, 95% CI 1.06–1.15; Stage III HR 1.81, 95% CI 1.76–1.86; Stage IV HR 4.05, 95% CI 3.96–4.15), and males were at higher risk than females (HR 1.21, 95% CI 1.19–1.24). Relative to ‘Other’ histologies, adenocarcinoma was associated with lower mortality hazards (HR 0.76, 95% CI 0.74–0.77).

**Fig. 2:**
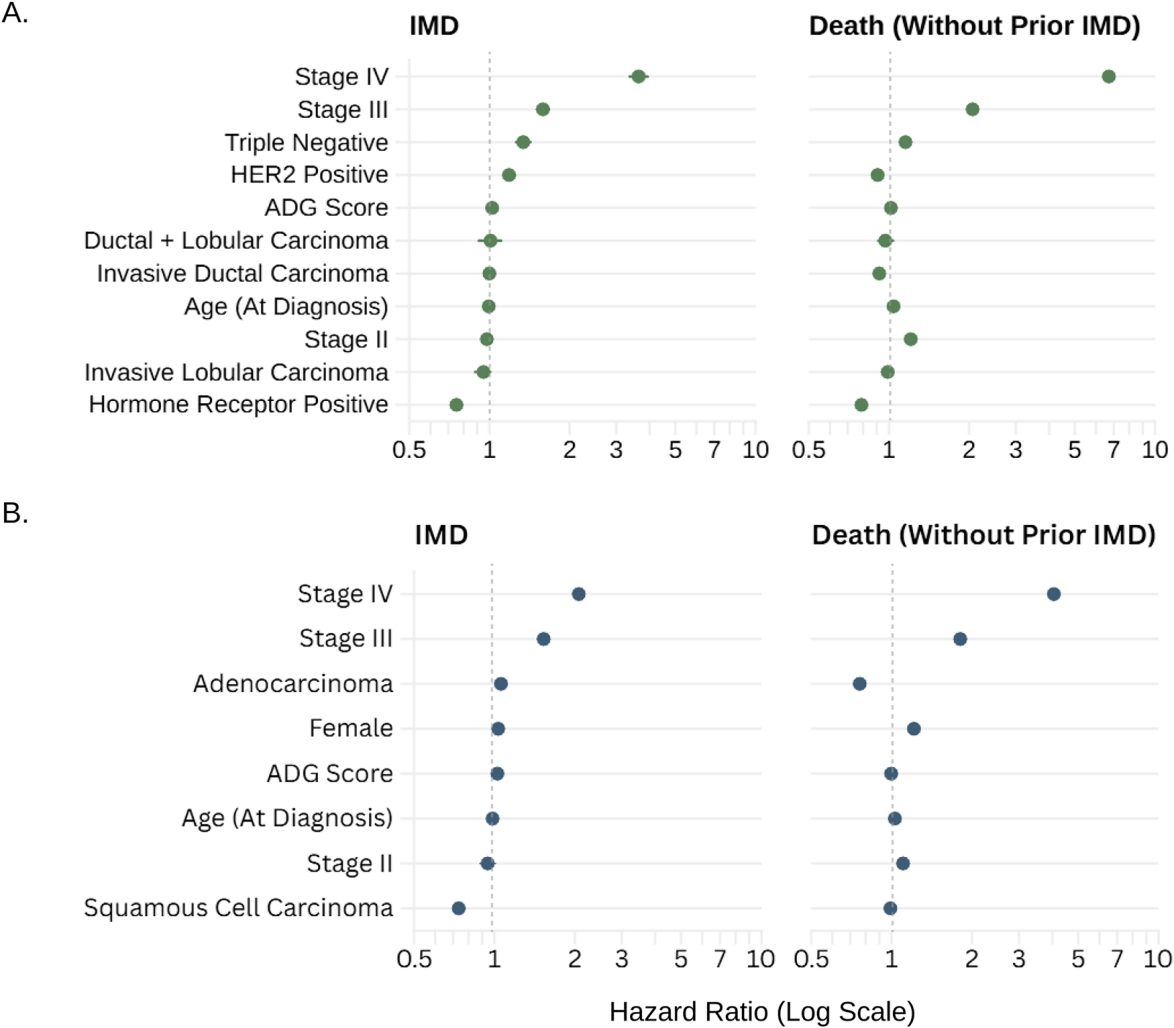
Cause-specific hazard ratios (HRs) for development of IMD and for death without prior IMD. **A** Breast cancer cohort. **B** Lung cancer cohort. Points show HRs from fully adjusted cause-specific Cox models on a log scale; the dashed vertical line marks HR = 1 and bars are 95% CIs. Reference levels for breast cancer: Stage I, “Other” histology. Reference levels for lung cancer: Stage I, “Other” histology, male.

Several covariates exhibited negligible or non-significant effects. Variables with 95% confidence intervals including 1.0, or statistically significant but clinically trivial magnitudes, included age and comorbidity status at diagnosis for both endpoints across both cancer types; histology in the breast-cancer IMD model; and sex in the lung-cancer IMD model. Figure 2 provides a complete visual summary of point estimates and 95% confidence intervals to facilitate appraisal of effect size and precision (see also Supplementary Tables S2, S3).

### Survival machine learning models

For each cancer type, we trained cause-specific machine-learning Cox models for two endpoints, development of IMD and death without prior IMD, on a 70% development set using five-fold cross-validation with Uno’s C-index as the optimization criterion. Feature definitions are detailed in Methods. Cause-specific hazards were then combined via the Aalen–Johansen estimator to yield patient-level cumulative-incidence predictions (AJ-CIF). All reported performance metrics were computed on the held-out 30% test set, with 95% confidence intervals by bootstrap.

#### Discrimination of cause-specific models

On the test set, discrimination for the IMD model was high in breast cancer (Uno’s C-index 0.95; CV: 0.94 ± 0.01) and moderate for the death model (0.82; CV: 0.82 ± 0.01). In lung cancer, Uno’s C-index was 0.88 for IMD (CV: 0.88 ± 0.03) and 0.78 for death (CV: 0.78 ± 0.03).

#### Time-dependent predictive accuracy and calibration of AJ-CIF

AJ-CIFs showed strong time-dependent performance at 1, 3, and 5 years. In the breast cancer cohort, AUROC(t) was 0.96, 0.97, and 0.96 (95% CIs: 0.95–0.97, 0.96–0.98, 0.95–0.96), and AUPRC(t) was 0.17, 0.53, and 0.63 (95% CIs: 0.16–0.20, 0.51–0.57, 0.61–0.68). The integrated Brier score (IBS) over 0–5 years was 0.009 (95% CIs: 0.008–0.010), a 35% reduction versus the null model. Calibration-in-the-large (CITL) was near zero across horizons (0.028, 0.016, 0.008; 95% CIs: 0.015–0.041, 0.006–0.026, 0.001–0.015), and the integrated calibration index (ICI) was 0.011 (95% CIs: 0.009–0.013).

**Fig. 3:**
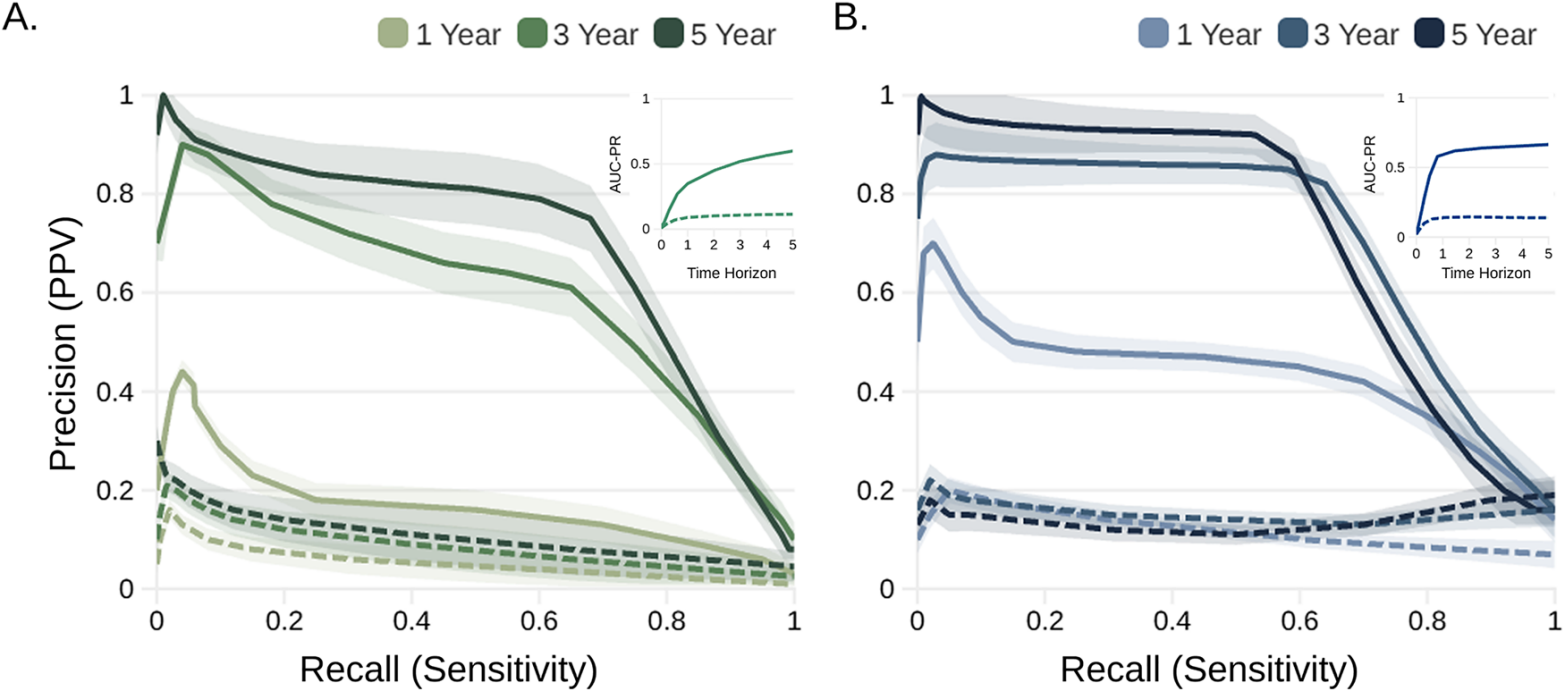
Time-dependent precision -recall performance of AJ-CIF predictions at 1, 3, and 5 years. **A** Breast cancer cohort. **B** Lung cancer cohort. Dashed lines represent age-and-stage-only baseline; shaded bands are 95% bootstrap CIs. Insets show the time-dependent area under the PR curve, AUPRC(t), across horizons.

In the lung cancer cohort, AUROC(t) was 0.89, 0.90, and 0.90 (95% CIs: 0.88–0.91, 0.89–0.91, 0.89–0.91), and AUPRC(t) was 0.37, 0.61, and 0.64 (95% CIs: 0.34–0.40, 0.58–0.64, 0.61–0.67). IBS was 0.060 (95% CIs: 0.056–0.064), a 40% reduction versus the null model. CITL moved toward zero over time (0.101 at 1 year to 0.012 at 5 years; 95% CIs: 0.085–0.117 and 0.004–0.020), and ICIs were modest across horizons (0.018, 0.027, 0.038; 95% CIs: 0.016–0.020, 0.025–0.030, 0.035–0.041). Time-dependent PR curves are shown in Fig. 3.

#### Clinical utility

**Fig. 4:**
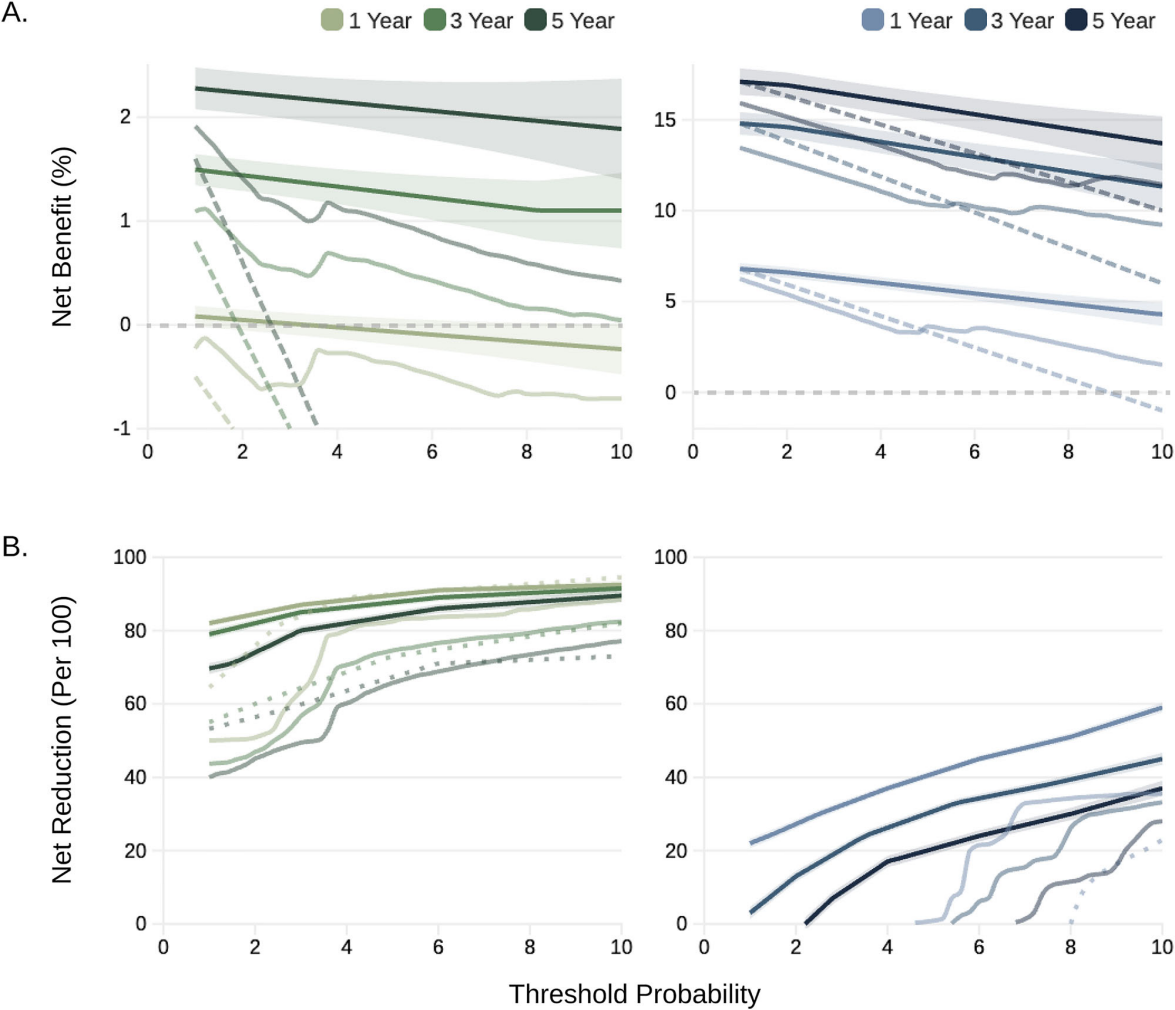
Clinical utility by decision-curve analysis for breast (green) and lung (blue) cancer. **A** Net benefit versus threshold probability (0–10%) for predicting IMD at 1, 3, and 5 years. Solid dark lines represent the complete AJ-CIF machine-learning models (tree-ensemble; all covariates), while solid faint lines represent the best-performing comparison models, the Standard AJ-CIF models (linear Cox; all covariates). the horizontal gray dashed line at 0 is the treat-none strategy; the colored dashed lines show the treat-all comparators. Shaded bands indicate 95% bootstrap CIs. **B** Net reduction in unnecessary interventions (per 100 patients) relative to treat-all across the same thresholds where a higher reduction is better. Dashed lines depict the treat-none reference; conventions otherwise as in (**A**).

**Fig. 5:**
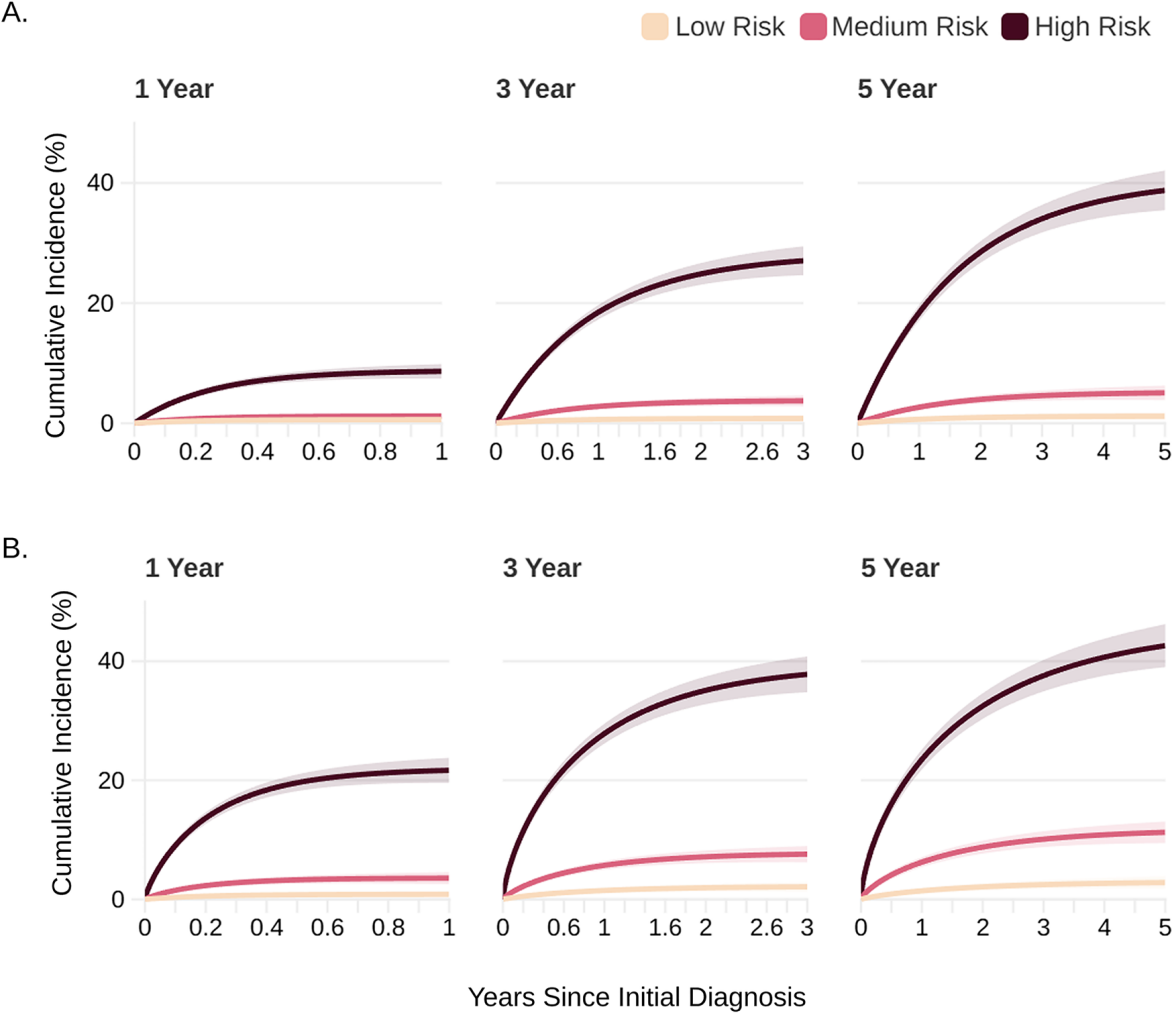
Observed cumulative incidence of IMD by model-based risk groups. **A** Breast cancer cohort. **B** Lung cancer cohort. Patients were stratified using predicted 5-year AJ-CIF into low (<5%), medium (5–10%), and high (>10%) risk groups. The breast cohort included 23,559 low-risk, 671 medium-risk, and 1595 high-risk patients; the lung cohort included 9838 low-risk, 1765 medium-risk, and 5575 high-risk patients. Curves show the observed cumulative incidence with 95% CIs. Risk groups are monotonically ordered at 1, 3, and 5 years and differences across groups were significant by Gray’s test at 1, 3, and 5 years (*p* < 0.001).

Decision-curve analysis across 0–10% thresholds showed the AJ-CIF models yielded higher net benefit than “treat none” or “treat all,” with clear separation at 3%, 5%, and 10% on the test set (Fig. 4). Using predicted 5-year AJ-CIF, patients were stratified into low (<5%), medium (5–10%), and high (>10%) risk groups (breast: low = 23,559; medium = 671; high = 1595; lung: low = 9838; medium = 1765; high = 5575). Observed CIFs were monotonically ordered with substantial absolute separation; Gray’s tests were significant at 1, 3, and 5 years for both cancers (*p* < 0.005; Fig. 5).

#### Comparison with baseline models

For completeness, we trained two comparator models under the same framework: an age-and-stage-only tree-ensemble baseline and a linear Cox model using the full covariate set. The age-and-stage baseline showed limited discrimination (Uno’s C-index, IMD: breast 0.79 [CV: 0.79 ± 0.01], lung 0.74 [CV: 0.74 ± 0.03]; death: breast 0.80 [CV: 0.80 ± 0.01], lung 0.72 [CV: 0.72 ± 0.03]). Discrimination improved with linear Cox models (Uno’s C-Index, IMD: breast 0.83 [CV: 0.83 ± 0.01], lung 0.75 [CV: 0.75 ± 0.01]; death: breast 0.80 [CV: 0.80 ± 0.02], lung 0.73 [CV: 0.73 ± 0.03]) but still underperformed the full tree-ensemble. Indeed, relative to linear Cox models, the full tree-ensemble increased Uno’s C-index for IMD by 15% (breast) and 17% (lung), with smaller gains for death (2% and 7%, respectively) (Supplementary Table S4). Furthermore, for clinical utility, we compared the full tree-ensemble against the best-performing comparator (linear Cox; all covariates); decision-curve analysis showed higher net benefit and net reduction across 1-, 3-, and 5-year horizons for the former set of models (Fig. 4; Supplementary Table S5).

### Feature importance of machine-learning models

**Fig. 6:**
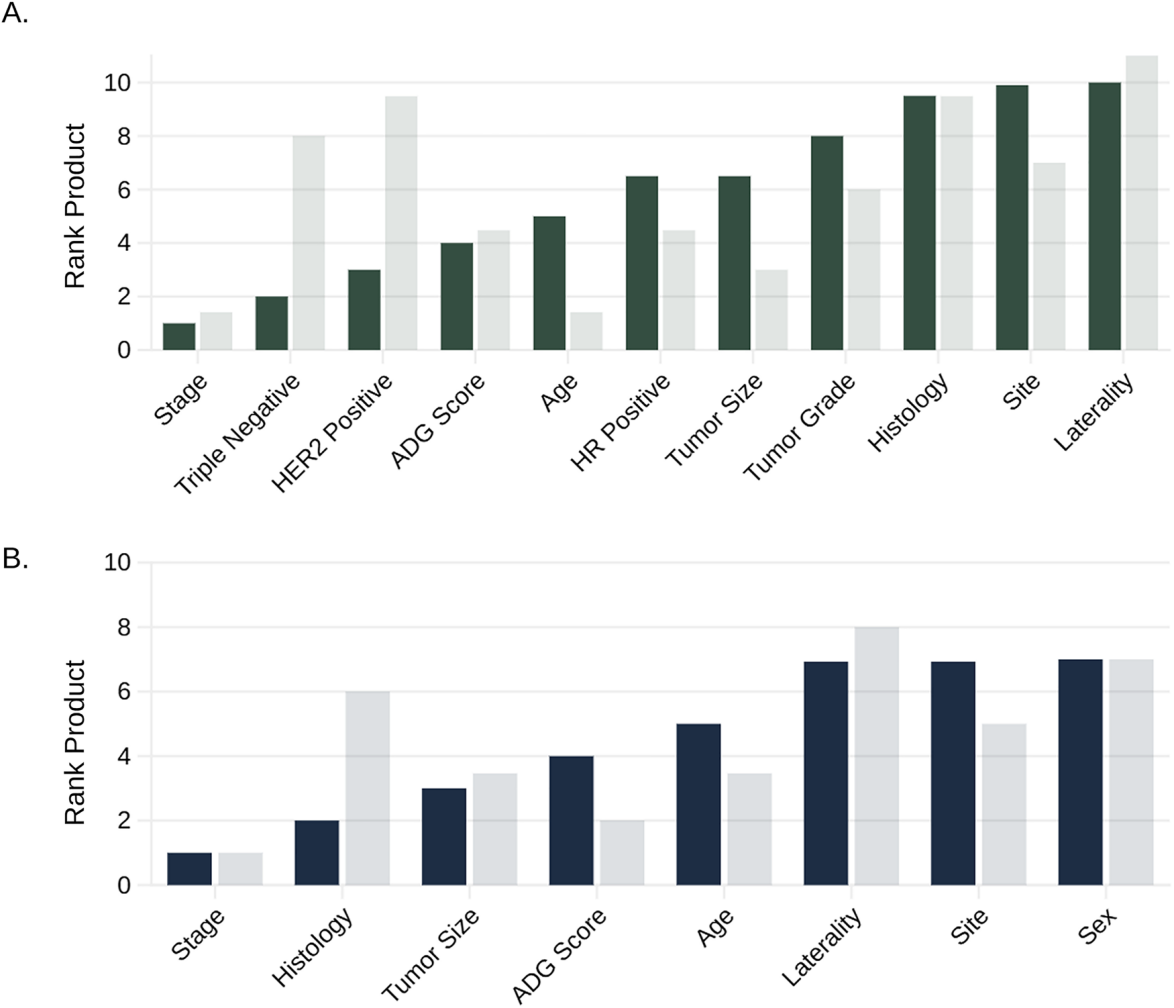
Feature importance by rank-product consensus. **A** Breast cancer cohort. **B** Lung cancer cohort. Bars show variable importance from the cause-specific machine-learning Cox models, computed as the rank product (geometric mean of SHAP and permutation ranks); smaller values indicate greater importance. Variables are ordered by increasing rank product (decreasing importance) for the IMD model. Opaque bars correspond to the IMD model; the adjacent translucent bars show the death-without-prior-IMD model for comparison.

We quantified feature contributions for each cause-specific machine-learning Cox model using two complementary approaches, Shapley Additive exPlanations (SHAP) and a permutation-based importance metric and combined them via a rank-product consensus (see Methods). The approaches were broadly concordant in identifying the most influential predictors across all four models, with only minor ordering differences. Figure 6 summarizes model-level importances; consensus ranks and the constituent SHAP and permutation orders are reported in Supplementary Tables S6–S9.

In breast cancer, the IMD model was driven primarily by stage, followed by triple-negative and HER2-positive status, mirroring the cause-specific Cox results in which advancing stage and these subtypes were associated with higher IMD hazard, whereas hormone-receptor–positive disease was protective. For death without prior IMD, stage remained the dominant contributor, tied with age at diagnosis, followed by tumor size. The two importance methods produced small rank reversals between age and stage, but both placed tumor size third.

In lung cancer, the IMD model highlighted stage, histology, and tumor size as the top contributors. For death, stage ranked first, followed by comorbidity status; thereafter, permutation ranked age ahead of tumor size, whereas SHAP ranked tumor size ahead of age.

## Discussion

In this study, we developed and validated competing-risk prediction models for intracranial metastatic disease (IMD) using a population-based administrative cohort. We trained cause-specific machine-learning Cox models for IMD and for death without prior IMD and combined them with the Aalen–Johansen estimator to generate patient-level cumulative-incidence predictions (AJ-CIFs) at clinically relevant horizons.

Across breast and lung cancers, the survival-based models achieved high discrimination with acceptable calibration and yielded clinically interpretable absolute-risk estimates over time. Discrimination of the cause-specific hazard models was strong by Uno’s C-index and horizon-specific performance of the models was robust at 3–5 years. Interestingly, performance at earlier horizons was attenuated, likely reflecting lower event prevalence early after diagnosis or potential etiologic heterogeneity whereby predictors of early-onset IMD differ from those of later metastasis^56–60^. Furthermore, overall prediction error showed meaningful reductions relative to an age-and-stage baseline and decision-curve analysis further demonstrated higher net benefit than treat-none or treat-all across clinically relevant thresholds. Collectively, these findings add to growing evidence that machine-learning approaches can enhance risk prediction in oncology^56–60^. Moreover, the models highlighted clinically coherent determinants of IMD risk in breast and lung cancer, aligning with, and extending, previous reports^35–38,61,62^.

In breast cancer, feature-importance analyses consistently prioritized stage and molecular subtype as the dominant contributors to IMD risk: rankings were stable across cross-validation folds and mirrored the stage-stratified separations at 1, 3, and 5 years. Cause-specific Cox models corroborated both direction and magnitude: hazards rose monotonically with stage, and HER2-positive and triple-negative subtypes carried higher IMD hazards, whereas hormone-receptor–positive disease was protective. These findings are consistent with prior reports linking subtype biology to metastatic propensity^39,40,42^. For death without prior IMD, the models again ranked stage first, followed by age and tumor size; however, while the cause-specific Cox estimates showed steep stage-dependent increases, the association with age was weak. The high model importance ranking of age, despite its modest hazard-based effect, likely reflects that models’ capacity to quantify multivariate contribution to risk stratification^35–38,61,62^.

In lung cancer, feature-importance analyses prioritized stage, histology, and tumor size as the leading contributors to IMD risk. These findings accord with prior work linking histotype to brain-metastatic propensity^16,18,32,60^. Cause-specific Cox estimates corroborated direction and magnitude, showing graded stage effects and lower IMD hazard for squamous cell carcinoma. For death without prior IMD, the models again ranked stage as the dominant driver, followed by comorbidity, with age and tumor size trailing (minor rank reversals between importance methods). The Cox models showed similar ordering, with steep stage-dependent increases in hazard and histology-associated differences aligning with reported patterns of mortality and metastatic behavior in lung cancer^32,41,50,63^.

While IMD is a relatively rare event in patients with metastatic cancer, its consequences are severe. Furthermore, the costs of default surveillance, both fiscally and practically, make global approaches to IMD surveillance unfeasible^64^. As such, there is significant clinical need for algorithms that can prospectively identify patients at increased risk for IMD who might benefit from clinical surveillance^7,17–20^. Our work describes the development and validation of ML models that can accurately predict the risk of IMD in patients with breast or lung cancer and potentially enable a more proactive, targeted and cost-effective approach to surveillance and management. Indeed, these models are designed to be used in a prospective clinical setting: they are based on features that may be readily collected by treating clinicians and require minimal computational power to deploy. This focus on practical application, along with model interpretability and an open-box framework, aims to build clinician trust and facilitate the integration of these predictive tools into routine practice^51,52^.

While our study presents promising results, its retrospective design and use of administrative health data may introduce biases related to data completeness, coding accuracy, and unmeasured confounds. For instance, absence of clinical and molecular granularity, particularly for lung cancer, where *EGFR*, *ALK*, and *ROS1* status were unavailable, limits adjustment for key biological drivers implicated in the metastatic process^40,41,43^. Similarly, our aggregation of small-cell lung cancer, a reported driver of IMD^45–47^, into a heterogenous ‘Other’ histology to maintain statistical stability due to sample-size limitations may attenuate histology-specific effects. Lastly, the models were trained on data from 2010–2023, a period marked by substantial advances in targeted therapy^3^. The increasing use of intracranially active targeted agents and immunotherapies likely altered baseline IMD risk over time. Our use of random rather than temporal data partitioning, while necessary to preserve adequate follow-up for long-horizon estimation, precludes assessment of such temporal drift. Consequently, future iterations should incorporate time-dependent treatment covariates and richer clinical and molecular features to ensure robust performance and sustained calibration in evolving therapeutic landscapes.

In conclusion, this study provides compelling evidence for the utility of ML to predict IMD in patients with breast or lung cancer. By providing accurate risk assessment and identifying key prognostic factors, our work can inform more effective and personalized cancer management strategies. Further validation across diverse populations and the integration of a broader range of data sources is needed to enhance model robustness and clinical utility. Notwithstanding, integrating such models into clinical practice holds significant potential for improving patient outcomes by facilitating early detection of IMD and reducing the overall burden of this serious complication.

## Supplementary information

Transparent Peer Review file

Supplemental Information

Description of Additional Supplementary files

Supplementary Data 1

Supplementary Data 2

Supplementary Data 3

Supplementary Data 4

Supplementary Data 5

Supplementary Data 6

## Data Availability

The dataset from this study is held securely in coded form at ICES, formerly known as the Institute for Clinical Evaluative Sciences. While legal data sharing agreements between ICES and data providers (e.g., healthcare organizations and government) prohibit ICES from making the dataset publicly available, access may be granted to those who meet pre-specified criteria for confidential access, available at www.ices.on.ca/DAS (email: das@ices.on.ca). The full dataset creation plan is available from the authors upon request, understanding that the computer programs may rely upon coding templates or macros that are unique to ICES and are therefore either inaccessible or may require modification. Public materials omit small-cell and other restricted granular codes in accordance with ICES privacy policy. The source data underlying all figures and graphs presented in this study are provided as Supplementary Data files accompanying this article.

## Code availability

The analytic code supporting this study is maintained within the secure, access-restricted computing environment and cannot be deposited in a public repository due to institutional privacy and security requirements. Code may be made available from the authors upon reasonable request to researchers, with the understanding that components may rely on institution-specific templates, macros, and infrastructure and may require modification for use in other settings.
