# Supplementary material for: Machine learning identifies prognosticators of intracranial metastatic disease in patients with breast or lung cancer
Source: Commun Med (Lond). 2026 Apr 23;6:364. doi: 10.1038/s43856-026-01609-3 (PMC13315275; doi:10.1038/s43856-026-01609-3)
Supplement: Supplementary file 2 — Supplemental Information [file 43856_2026_1609_MOESM2_ESM.pdf]

## Supplementary Tables

**Supplementary Table S1. Cumulative incidence of IMD and death without prior IMD at 1, 3, and 5 years in breast and lung cancer cohorts.** CIFs calculated using Aalen-Johansen estimator. Rates are per 100 person-years (PY) with 95% CIs.

| | Breast Cancer | Lung Cancer |
|---|---|---|
| Cumulative incidence of IMD at 1 year, % (95% CI) | 0.6 (0.5–0.6) | 6.6 (6.4–6.8) |
| Cumulative incidence of IMD at 3 years, % (95% CI) | 1.8 (1.7–1.9) | 10.2 (9.9–10.4) |
| Cumulative incidence of IMD at 5 years, % (95% CI) | 2.6 (2.5–2.7) | 11.0 (10.8–11.3) |
| Cumulative incidence of death (no prior IMD) at 1 year, % (95% CI) | 2.9 (2.8–3.0) | 42.8 (42.4–43.3) |
| Cumulative incidence of death (no prior IMD) at 3 years, % (95% CI) | 7.9 (7.7–8.1) | 58.4 (58.0–58.8) |
| Cumulative incidence of death (no prior IMD) at 5 years, % (95% CI) | 12.5 (12.3–12.7) | 64.3 (63.9–64.7) |
| IMD incidence rate, per 100 PY (95% CI) | 0.45 (0.43–0.47) | 4.54 (4.43–4.65) |
| Death incidence rate (no prior IMD), per 100 PY (95% CI) | 2.68 (2.64–2.73) | 27.61 (27.34–27.88) |

**Supplementary Table S2. Breast cancer: cause-specific Cox models for IMD and for death without prior IMD.** Values are hazard ratios (HR) with 95% confidence intervals; rows are ordered by IMD HR from highest to lowest. Age effect per 1 year; ADG per unit.

| Variable | IMD (HR, 95% CI) | Death without IMD (HR, 95% CI) |
|---|---|---|
| Stage IV (ref: Stage I) | 3.64 (3.352–3.953) | 6.709 (6.4–7.032) |
| Stage III (ref: Stage I) | 1.589 (1.503–1.681) | 2.063 (1.987–2.141) |
| Triple negative | 1.34 (1.255–1.431) | 1.154 (1.099–1.213) |
| HER2 positive | 1.185 (1.123–1.252) | 0.907 (0.871–0.944) |
| ADG score | 1.024 (1.023–1.026) | 1.018 (1.017–1.019) |
| Ductal + lobular carcinoma (ref: “Other” histology) | 1.01 (0.91–1.11) | 0.97 (0.91–1.04) |
| Invasive ductal carcinoma (ref: “Other” histology) | 1.00 (0.96–1.05) | 0.92 (0.89–0.94) |
| Age at diagnosis (per year) | 0.994 (0.992–0.995) | 1.041 (1.040–1.042) |
| Stage II (ref: Stage I) | 0.978 (0.94–1.018) | 1.208 (1.174–1.242) |
| Invasive lobular carcinoma (ref: “Other” histology) | 0.95 (0.88–1.01) | 0.99 (0.94–1.04) |
| Hormone-receptor positive | 0.752 (0.717–0.789) | 0.789 (0.761–0.817) |

**Supplementary Table S3. Lung cancer: cause-specific Cox models for intracranial metastatic disease (IMD) and for death without prior IMD.** Conventions are as Supplementary Table S2.

| Variable | IMD (HR, 95% CI) | Death without IMD (HR, 95% CI) |
|---|---|---|
| Stage IV (ref: Stage I) | 2.069 (1.989–2.153) | 4.055 (3.963–4.15) |
| Stage III (ref: Stage I) | 1.528 (1.461–1.597) | 1.809 (1.761–1.858) |
| Adenocarcinoma (ref: “Other” histology) | 1.058 (1.019–1.099) | 0.76 (0.74–0.77) |
| Female (ref: Male) | 1.033 (0.996–1.072) | 1.213 (1.19–1.236) |
| ADG score | 1.026 (1.024–1.027) | 0.997 (0.997–0.998) |
| Age at diagnosis (per year) | 0.984 (0.983–0.986) | 1.029 (1.028–1.03) |
| Stage II (ref: Stage I) | 0.943 (0.885–1.005) | 1.104 (1.064–1.145) |
| Squamous cell carcinoma (ref: “Other” histology) | 0.735 (0.699–0.773) | 0.99 (0.97–1.02) |

**Supplementary Table S4a. Discrimination and overall error for AJ-CIF models (30% held-out test set).** Model conventions as follows: Breast (all features; nonlinear tree-based model), Breast Baseline (age + stage; nonlinear tree-based model), Breast Standard (all features; linear Cox model), Lung, Lung Baseline, and Lung Standard. Values are point estimates with 95% bootstrap CIs. Lower is better for IBS. Abbreviations: AUROC, area under the receiver operating characteristic; AUPRC, area under the precision–recall curve; IBS, integrated Brier score.

| Metric | Breast | Breast Baseline | Breast Standard | Lung | Lung Baseline | Lung Standard |
|---|---|---|---|---|---|---|
| AUROC, 1 y | 0.96 (0.91–0.99) | 0.85 (0.80–0.90) | 0.84 (0.82–0.86) | 0.89 (0.84–0.94) | 0.75 (0.68–0.82) | 0.72 (0.70–0.74) |
| AUROC, 3 y | 0.97 (0.94–1.0) | 0.84 (0.79–0.89) | 0.87 (0.86–0.88) | 0.90 (0.87–0.93) | 0.78 (0.71–0.85) | 0.75 (0.74–0.77) |
| AUROC, 5 y | 0.96 (0.93–0.99) | 0.82 (0.77–0.87) | 0.87 (0.86–0.88) | 0.90 (0.87–0.93) | 0.77 (0.70–0.84) | 0.75 (0.74–0.76) |
| AUPRC, 1 y | 0.17 (0.12–0.22) | 0.049 (0.020–0.078) | 0.05 (0.03–0.06) | 0.37 (0.31–0.43) | 0.08 (0.051–0.11) | 0.17 (0.15–0.19) |
| AUPRC, 3 y | 0.53 (0.45–0.61) | 0.10 (0.051–0.15) | 0.20 (0.17–0.23) | 0.61 (0.53–0.69) | 0.11 (0.061–0.16) | 0.29 (0.27–0.31) |
| AUPRC, 5 y | 0.63 (0.55–0.71) | 0.11 (0.061–0.16) | 0.15 (0.11–0.19) | 0.64 (0.56–0.72) | 0.087 (0.058–0.12) | 0.31 (0.29–0.33) |
| IBS (0–5 y) | 0.0090 (0.0050–0.013) | 0.013 (0.0080–0.018) | 0.01 (0.01–0.01) | 0.060 (0.036–0.084) | 0.10 (0.061–0.14) | 0.080 (0.070–0.080) |
| IBS reduction (vs null) | 0.0042 (35%) | 0.0011 (8.0%) | 0.0041 (29%) | 0.040 (40%) | 0.0050 (5.0%) | 0.020 (20%) |

**Supplementary Table S4b. Calibration metrics for AJ-CIF models (30% held-out test set).** Model conventions are as Supplementary Table S4a. Values are estimates with 95% bootstrap CIs. Lower metric scores are better**.** Abbreviations: CITL, calibration-in-the-large; ICI, integrated calibration index; E50 and E90, median and 90th-percentile absolute calibration error, respectively.

| Metric | Breast | Breast Baseline | Breast Standard | Lung | Lung Baseline | Lung Standard |
|---|---|---|---|---|---|---|
| CITL, 1 y | 0.028 (0.014–0.042) | 0.031 (0.016–0.046) | 0.029 (0.014–0.043) | 0.10 (0.052–0.15) | 0.10 (0.053–0.15) | 0.10 (0.052–0.15) |
| CITL, 3 y | 0.016 (0.008–0.024) | 0.019 (0.010–0.028) | 0.017 (0.0085–0.025) | 0.030 (0.015–0.045) | 0.034 (0.017–0.051) | 0.032 (0.016–0.048) |
| CITL, 5 y | 0.008 (0.004–0.012) | 0.011 (0.006–0.016) | 0.0087 (0.0045–0.013) | 0.012 (0.006–0.018) | 0.014 (0.007–0.021) | 0.013 (0.0065–0.020) |
| ICI, 1 y | 0.011 (0.006–0.016) | 0.020 (0.010–0.030) | 0.014 (0.007–0.020) | 0.018 (0.009–0.027) | 0.039 (0.020–0.058) | 0.029 (0.014–0.043) |
| ICI, 3 y | 0.011 (0.006–0.016) | 0.021 (0.011–0.031) | 0.013 (0.007–0.020) | 0.027 (0.014–0.040) | 0.037 (0.019–0.055) | 0.031 (0.016–0.048) |
| ICI, 5 y | 0.011 (0.006–0.016) | 0.021 (0.011–0.031) | 0.013 (0.007–0.020) | 0.038 (0.019–0.057) | 0.042 (0.021–0.063) | 0.040 (0.020–0.061) |
| E50, 1 y | 0.001 (0.0–0.002) | 0.003 (0.001–0.005) | 0.0014 (0.00025–0.0028) | 0.011 (0.005–0.017) | 0.024 (0.010–0.038) | 0.018 (0.008–0.028) |
| E50, 3 y | 0.002 (0.001–0.003) | 0.002 (0.001–0.003) | 0.0020 (0.0010–0.0030) | 0.017 (0.007–0.027) | 0.022 (0.009–0.035) | 0.019 (0.008–0.031) |
| E50, 5 y | 0.002 (0.001–0.003) | 0.003 (0.001–0.005) | 0.0023 (0.0010–0.0035) | 0.032 (0.013–0.051) | 0.022 (0.009–0.035) | 0.027 (0.011–0.043) |

| | | | | | | |
|---|---|---|---|---|---|---|
| E90, 1 y | 0.014 (0.004–0.024) | 0.037 (0.012–0.062) | 0.019 (0.006–0.034) | 0.039 (0.012–0.066) | 0.074 (0.023–0.125) | 0.057 (0.018–0.096) |
| E90, 3 y | 0.012 (0.004–0.020) | 0.035 (0.011–0.059) | 0.018 (0.006–0.030) | 0.065 (0.020–0.110) | 0.067 (0.021–0.113) | 0.066 (0.020–0.112) |
| E90, 5 y | 0.012 (0.004–0.020) | 0.029 (0.009–0.049) | 0.016 (0.005–0.028) | 0.093 (0.029–0.157) | 0.074 (0.023–0.125) | 0.084 (0.026–0.142) |

**Supplementary Table S5a. Decision-curve analysis net benefit at selected risk-threshold probabilities.** Values are net benefit (vs treat-none) point estimates with 95% bootstrap CIs. Model conventions are as Supplementary Table S4a.

| Time (y) | Threshold ($p_t$) | Breast | Breast Baseline | Breast Standard | Lung | Lung Baseline | Lung Standard |
|---|---|---|---|---|---|---|---|
| 1 | 0.03 | 0.0018 (0.0013–0.0023) | -0.0061 (-0.0081–-0.0041) | -0.0022 (-0.0029–-0.0014) | 0.062 (0.053–0.071) | 0.050 (0.041–0.059) | 0.056 (0.048–0.064) |
| 1 | 0.05 | 0.00076 (0.00026–0.0013) | -0.0039 (-0.0056–-0.0022) | -0.0016 (-0.0021–-0.0010) | 0.054 (0.046–0.062) | 0.040 (0.033–0.047) | 0.047 (0.039–0.055) |
| 1 | 0.1 | -0.0012 (-0.0017–-0.00069) | -0.0073 (-0.011–-0.0039) | -0.0043 (-0.0061–-0.0024) | 0.041 (0.034–0.048) | 0.016 (0.012–0.020) | 0.029 (0.022–0.035) |
| 3 | 0.03 | 0.014 (0.011–0.017) | 0.0051 (0.0034–0.0068) | 0.0095 (0.0067–0.012) | 0.14 (0.12–0.16) | 0.13 (0.11–0.15) | 0.14 (0.12–0.15) |
| 3 | 0.05 | 0.013 (0.0094–0.017) | 0.0054 (0.0035–0.0073) | 0.0092 (0.0065–0.012) | 0.13 (0.11–0.15) | 0.11 (0.091–0.13) | 0.12 (0.10–0.14) |
| 3 | 0.1 | 0.011 (0.0075–0.015) | 0.00019 (-0.00031–0.00069) | 0.0056 (0.0035–0.0077) | 0.11 (0.094–0.13) | 0.10 (0.080–0.12) | 0.11 (0.086–0.12) |
| 5 | 0.03 | 0.021 (0.016–0.026) | 0.011 (0.0078–0.014) | 0.016 (0.012–0.020) | 0.16 (0.14–0.18) | 0.16 (0.14–0.18) | 0.16 (0.14–0.18) |
| 5 | 0.05 | 0.020 (0.015–0.025) | 0.0099 (0.0067–0.013) | 0.015 (0.011–0.019) | 0.15 (0.13–0.17) | 0.14 (0.12–0.16) | 0.15 (0.13–0.16) |
| 5 | 0.1 | 0.018 (0.013–0.023) | 0.0041 (0.0026–0.0056) | 0.011 (0.0073–0.015) | 0.14 (0.12–0.16) | 0.13 (0.11–0.15) | 0.14 (0.11–0.16) |

**Supplementary Table S5b. Decision-curve analysis net reduction at selected risk-threshold probabilities.** Values are interventions avoided per 100 (vs treat-all) point estimates with 95% bootstrap CIs. Model conventions are as Supplementary Table S4a.

| Time (y) | Threshold ($p_t$) | Breast | Breast Baseline | Breast Standard | Lung | Lung Baseline | Lung Standard |
|---|---|---|---|---|---|---|---|
| 1 | 0.03 | 88 (80–96) | 63 (57–69) | 76 (69–82) | 39 (36–42) | 0.30 (-1.7–2.3) | 20 (18–22) |
| 1 | 0.05 | 91 (83–99) | 82 (73–91) | 86 (78–95) | 47 (43–51) | 20 (18–22) | 34 (30–37) |
| 1 | 0.1 | 94 (85–103) | 88 (78–98) | 91 (82–100) | 59 (54–64) | 36 (33–39) | 48 (43–52) |
| 3 | 0.03 | 85 (78–92) | 57 (51–63) | 71 (64–78) | 20 (18–22) | 0.48 (-1.5–2.5) | 10 (8.2–12) |
| 3 | 0.05 | 88 (80–96) | 74 (66–82) | 81 (73–89) | 30 (27–33) | 3.2 (1.2–5.2) | 17 (15–19) |
| 3 | 0.1 | 92 (83–101) | 82 (73–91) | 87 (78–96) | 45 (41–49) | 34 (30–38) | 40 (35–44) |
| 5 | 0.03 | 80 (73–87) | 47 (42–52) | 64 (57–70) | 7.2 (5.2–9.2) | 0.62 (-1.4–2.6) | 3.9 (1.9–5.9) |
| 5 | 0.05 | 85 (78–92) | 66 (58–74) | 76 (68–83) | 21 (19–23) | -2.0 (-4.0–0) | 9.5 (7.5–12) |
| 5 | 0.1 | 90 (81–99) | 77 (67–87) | 84 (75–92) | 37 (34–40) | 29 (26–32) | 33 (29–37) |

**Supplementary Table S6. Breast cancer IMD model: feature-importance ranks from SHAP and permutation with consensus ranks.** Ranks are ordered by IMD importance; lower ranks indicate greater importance. Columns show SHAP rank (with mean |SHAP|), permutation rank (normalized Δ prediction), the arithmetic mean of the two ranks ("Average rank"), and their geometric mean ("Rank product").

| Consensus Rank | Feature | SHAP Rank (Mean \|SHAP\|) | Permutation Rank (Δ Prediction) | Average Rank | Rank Product |
|---|---|---|---|---|---|
| 1 | Stage at diagnosis | 1 (1.9) | 1 (41) | 1 | 1 |
| 2 | Triple-negative | 2 (0.76) | 2 (18) | 2 | 2 |
| 3 | HER2 status | 3 (0.46) | 3 (15) | 3 | 3 |
| 4 | Comorbidity (ADG) score | 4 (0.41) | 4 (8.6) | 4 | 4 |
| 5 | Age at diagnosis | 5 (0.26) | 5 (7.2) | 5 | 5 |
| 6 | Hormone-receptor status | 7 (0.15) | 6 (2.5) | 6.5 | 6.5 |
| 7 | Tumor size | 6 (0.23) | 7 (1.7) | 6.5 | 6.5 |
| 8 | Tumor grade | 8 (0.14) | 8 (1.6) | 8 | 8 |
| 9 | Histology subtype | 9 (0.11) | 10 (1.6) | 9.5 | 9.5 |
| 10 | Primary site | 11 (0.083) | 9 (1.6) | 10 | 9.9 |
| 11 | Laterality | 10 (0.099) | 11 (1) | 10 | 10 |

**Supplementary Table S7. Breast cancer death model: feature-importance ranks from SHAP and permutation with consensus ranks.** Conventions are as Supplementary Table S5.

| Consensus Rank | Feature | SHAP Rank (Mean \|SHAP\|) | Permutation Rank (Δ Prediction) | Average Rank | Rank Product |
|---|---|---|---|---|---|
| 1 | Age at diagnosis | 1 (0.4) | 2 (24) | 1.5 | 1.414 |
| 1 | Stage at diagnosis (AJCC) | 2 (0.36) | 1 (31.6) | 1.5 | 1.414 |
| 2 | Tumor size | 3 (0.33) | 3 (16.4) | 3.0 | 3.0 |
| 3 | Comorbidity (ADG) score | 5 (0.085) | 4 (10.2) | 4.5 | 4.472 |
| 3 | Hormone-receptor (HR) status | 4 (0.24) | 5 (3.6) | 4.5 | 4.472 |
| 4 | Tumor grade | 6 (0.068) | 6 (3.38) | 6.0 | 6.0 |
| 5 | Primary site | 7 (0.056) | 7 (2.62) | 7.0 | 7.0 |
| 6 | Triple-negative | 8 (0.053) | 8 (2.51) | 8.0 | 8.0 |
| 7 | HER2 status | 9 (0.036) | 10 (1.85) | 9.5 | 9.487 |
| 7 | Histology subtype | 10 (0.031) | 9 (2.07) | 9.5 | 9.487 |
| 8 | Laterality | 11 (0.026) | 11 (1.75) | 11.0 | 11.0 |

**Supplementary Table S8. Lung cancer IMD model: feature-importance ranks from SHAP and permutation with consensus ranks.** Conventions are as Supplementary Table S5.

| Consensus Rank | Feature | SHAP Rank (Mean \|SHAP\|) | Permutation Rank (Δ Prediction) | Average Rank | Rank Product |
|---|---|---|---|---|---|
| 1 | Stage at diagnosis (AJCC) | 1 (0.89) | 1 (34.3) | 1.0 | 1.0 |
| 2 | Histology subtype | 2 (0.64) | 2 (19.6) | 2.0 | 2.0 |
| 3 | Tumor size | 3 (0.26) | 3 (12.7) | 3.0 | 3.0 |
| 4 | Comorbidity (ADG) score | 4 (0.2) | 4 (8.58) | 4.0 | 4.0 |
| 5 | Age at diagnosis | 5 (0.17) | 5 (8.21) | 5.0 | 5.0 |
| 6 | Laterality | 6 (0.14) | 8 (3.31) | 7.0 | 6.928 |
| 6 | Primary site | 8 (0.065) | 6 (4.66) | 7.0 | 6.928 |
| 7 | Sex | 7 (0.088) | 7 (4.16) | 7.0 | 7.0 |

**Supplementary Table S9. Lung cancer death model: feature-importance ranks from SHAP and permutation with consensus ranks.** Conventions are as Supplementary Table S5.

| Consensus Rank | Feature | SHAP Rank (Mean \|SHAP\|) | Permutation Rank (Δ Prediction) | Average Rank | Rank Product |
|---|---|---|---|---|---|
| 1 | Stage at diagnosis (AJCC) | 1 (0.64) | 1 (46.7) | 1.0 | 1.0 |
| 2 | Comorbidity (ADG) score | 2 (0.33) | 2 (12.5) | 2.0 | 2.0 |
| 3 | Age at diagnosis | 4 (0.21) | 3 (10.5) | 3.5 | 3.464 |
| 3 | Tumor size | 3 (0.22) | 4 (9.91) | 3.5 | 3.464 |
| 4 | Primary site | 5 (0.17) | 5 (7.06) | 5.0 | 5.0 |
| 5 | Histology subtype | 6 (0.17) | 6 (5.47) | 6.0 | 6.0 |
| 6 | Sex | 7 (0.12) | 7 (3.98) | 7.0 | 7.0 |
| 7 | Laterality | 8 (0.11) | 8 (3.87) | 8.0 | 8.0 |

**Supplementary Table S10. Demographic overview of breast cancer cohort. Numbers represent the pre-imputation distribution.** Values are mean (±SD) or n (%). Abbreviations: ICD-10, International Classification of Diseases 10th Revision; NOS, not otherwise specified.

| Feature | M (±SD) / n (%) |
|---|---|
| Age at diagnosis (years) | 62 (±14) |
| Tumor size (mm) | 25 (±22) |
| Laterality (Left : Right) | 43,690 (51%) : 42,392 (49%) |
| **Site (ICD-10 code)** | |
| Central portion of breast (C50.1) | 4,511 (5%) |
| Upper-inner quadrant (C50.2) | 11,966 (14%) |
| Lower-inner quadrant (C50.3) | 5,305 (6%) |
| Upper-outer quadrant (C50.4) | 32,739 (38%) |
| Lower-outer quadrant (C50.5) | 8,412 (10%) |
| Overlapping lesion of breast (C50.8) | 15,036 (18%) |
| Breast, NOS (C50.9) | 6,259 (7%) |
| Other (C50.0, C50.6) | 1,854 (3%) |
| **Histology (ICD-O-3 Code)** | |
| Infiltrating duct (85003) | 66,169 (77%) |
| Lobular (85203) | 7,153 (8%) |
| Infiltrating duct + lobular (85223) | 4,158 (5%) |
| Other (NA) | 8,602 (10%) |
| **Stage** | |
| I | 44,926 (52%) |
| II | 27,125 (31%) |
| III | 9,970 (12%) |
| IV | 3,991 (5%) |
| **Grade** | |
| 1 | 15,166 (18%) |
| 2 | 46,763 (54%) |
| 3 | 24,101 (28%) |
| 4 | 52 (0%) |
| **Receptor status** | |

| | |
|---|---|
| ER-positive | 72,673 (84%) |
| PR-positive | 64,495 (75%) |
| HER2-positive | 11,148 (13%) |
| Triple-negative | 7,123 (8%) |

**Supplementary Table S11. Demographic overview of lung cancer cohort. Numbers represent the pre-imputation distribution.** Conventions are as Supplementary Table S10.

| **Feature** | **M (±SD) / n (%)** |
|---|---|
| Age at diagnosis (years) | 70 (±10) |
| Tumor size (mm) | 43 (±27) |
| Sex (Female : Male) | 28,558 (50%) : 28,701 (50%) |
| Laterality (Left : Right) | 21,891 (38%) : 35,368 (62%) |
| **Site (ICD-10 Code)** | |
| Upper lobe, bronchus or lung (C34.1) | 31,529 (55%) |
| Middle lobe, bronchus or lung (C34.2) | 2,679 (5%) |
| Lower lobe, bronchus or lung (C34.3) | 16,074 (28%) |
| Overlapping lesion of bronchus and lung (C34.8) | 288 (1%) |
| Bronchus or lung, NOS (C34.9) | 6,689 (12%) |
| **Histology (ICD-O-3 Code)** | |
| Adenocarcinoma (81403) | 21,900 (38%) |
| Squamous cell carcinoma (80703) | 10,788 (19%) |
| Other (NA) | 24,571 (43%) |
| **Stage** | |
| I | 13,982 (24%) |
| II | 5,324 (9%) |
| III | 12,311 (22%) |
| IV | 25,641 (45%) |

**Supplementary Table S12. ADG categories and weights used in this study to compute the ADG score.** Weights reproduced from Austin & van Walraven[62].

| ADG Category | Weight |
|---|---|
| Time Limited: Minor | 0 |
| Time Limited: Minor-Primary Infections | 0 |
| Time Limited: Major | 6 |
| Time Limited: Major-Primary Infections | 4 |
| Allergies | -6 |
| Asthma | 0 |
| Likely to Recur: Discrete | 0 |
| Likely to Recur: Discrete-Infections | 0 |
| Likely to Recur: Progressive | 8 |
| Chronic Medical: Stable | 4 |
| Chronic Medical: Unstable | 12 |
| Chronic Specialty: Stable-Orthopedic | -3 |
| Chronic Specialty: Stable-Ear, Nose, Throat | 0 |
| Chronic Specialty: Stable-Eye | 3 |
| Chronic Specialty: Unstable-Orthopedic | -2 |
| Chronic Specialty: Unstable-Ear, Nose, Throat | -4 |
| Chronic Specialty: Unstable-Eye | 1 |
| Dermatologic | -4 |
| Injuries/Adverse Effects: Minor | -1 |
| Injuries/Adverse Effects: Major | 2 |
| Psychosocial: Time Limited, Minor | -1 |
| Psychosocial: Recurrent or Persistent, Stable | -3 |
| Psychosocial: Recurrent or Persistent, Unstable | 16 |
| Signs/Symptoms: Minor | 3 |
| Signs/Symptoms: Uncertain | 2 |
| Signs/Symptoms: Major | 2 |
| Discretionary | -2 |
| See and Reassure | 1 |

| | |
|---|---|
| Prevention/Administrative | -2 |
| Malignancy | 13 |
| Pregnancy | -19 |
| Dental | -1 |

**Supplementary Table S13. Feature set and missingness for breast and lung cancer cohorts.** Entries show percent missing. A dash (—) denotes features absent in that cohort. Abbreviations: ADG, Aggregated Diagnosis Groups.

| Feature | Breast Cohort (% Missing) | Lung Cohort (% Missing) |
|---|---|---|
| ADG score | 0 | 0 |
| Age | 0 | 0 |
| HER2 status | 20 | — |
| Histology | 0 | 0 |
| HR status | 0 | — |
| Laterality | 2 | 6 |
| Sex | — | 0 |
| Stage | 1 | 1 |
| Triple-negative status | 0 | — |
| Tumor Grade | 11 | - |
| Tumor Size | 3 | 4 |
