# Supplementary material for: Machine learning identifies prognosticators of intracranial metastatic disease in patients with breast or lung cancer
Source: Commun Med (Lond). 2026 Apr 23;6:364. doi: 10.1038/s43856-026-01609-3 (PMC13315275; doi:10.1038/s43856-026-01609-3)
Supplement: Supplementary file 3 — Description of Additional Supplementary files [file 43856_2026_1609_MOESM3_ESM.docx]

Description of Additional Supplementary Files

File name: Supplementary Data 1-6

Description: Source data behind the graphs in the paper
