# Supplementary material for: Machine learning identifies prognosticators of intracranial metastatic disease in patients with breast or lung cancer
Source: Commun Med (Lond). 2026 Apr 23;6:364. doi: 10.1038/s43856-026-01609-3 (PMC13315275; doi:10.1038/s43856-026-01609-3)
Supplement: Supplementary file 1 — Transparent Peer Review file [file 43856_2026_1609_MOESM1_ESM.pdf]

nature portfolio

Peer Review File

Machine learning identifies prognosticators of intracranial metastatic disease in patients with breast or lung cancer

Corresponding Author: Dr Sunit Das

This file contains all reviewer reports in order by version, followed by all author rebuttals in order by version.

Version 0:

Reviewer comments:

Reviewer #1

(Remarks to the Author)
This is an important study on building a prediction model for intracranial metastasis in breast and lung cancer. Although prediction models are not new, this study is notable for the size of its cohort and inclusion of two cancer types. Some methodological clarifications could strengthen this manuscript.

Major comments:
1. The outcomes for the classification model are not clearly described. Specifically, it is unclear what proportion of the cohort died before they could develop a brain metastasis, and whether or how the competing risk of death was accounted for. Please describe.
2. Please include a table of the rate of missingness in predictor variables in both cohorts.
3. Please include a specific definition of time zero (presumably date of initial diagnosis?) and how the outcome of brain metastasis was defined.
4. Systemic treatment, specifically use of intra-cranially penetrant vs not, can significantly influence the development of brain metastases. Use has also changed significantly across the time period in the study (2010 - 2023). For example, the intracranially penetrant EGFR TKI osimertinib was introduced in 2015. It is unclear whether a model trained on data from a past era will continue to hold validity as systemic therapies change. Please justify a) using a validation test set that is randomly sampled across all time periods, instead of one split by time and b) why initial choice of systemic treatments are excluded from the model; or include as a limitation.

Minor comments:
1. Small cell lung cancer histology is known to be significantly associated with increased risk of brain metastasis (https://www.jtocrr.org/article/S2666-3643(22)00054-6/fulltext). Justify why it is grouped under "Other" histology or separate the histology out.
2. Please include a table of the specific " 34 comorbidity-based features" mentioned in line 311 of the methods.

Reviewer #2

(Remarks to the Author)
Using a large database, the authors develop machine learning models to predict intracranial metastatic disease (IMD) for breast cancer and lung cancer using EHR-related tabular data. I find the application to be clinically significant and well motivated, but I have several major concerns regarding the analysis.

1. The studied problem - risk of developing IMD over time - is best formulated as a time-to-event problem, where each patient is ultimately a) censored (according to latest follow up), b) experiences a competing risk (death), or c) experiences IMD. However, all the analysis is performed as a binary task (IMD event or not) and I can't find any discussion or reporting of follow-up times. This introduces risk of bias as patients may be assigned an outcome of "0" just because they had short follow up times. Even if the model is developed in the purely binary setting, it is important to evaluate it using time-to-event metrics such as the concordance index.

2. The ML model performance is currently lacking baselines. For instance, what is the predictive performance of stage alone? Table 1 reports a chi-squared p-value for univariates, but the actual predictive performance is not reported, making it

unclear how much better the ML model is than baselines.

3. The training/validation/test splitting is currently unclear. The methods first state that "The dataset was first split into training (49%), validation (21%), and test (30%) sets". Later, it is stated "To ensure robust model evaluation and mitigate overfitting, a five-fold cross-validation (CV) strategy was adopted", and "Following model training on the validation set within each CV fold...". Separately, is there a reason for the non-round numbers of 49% and 21%?

4. The main text describes the clinical features included in the model such as stage, histology, etc. However, in the methods, it is also stated that 34 ADG features are also included in the model, which are not described in the main text besides brief mention in a figure caption. Given the large number of features, it's important to describe these in the main text and what they mean, especially given the paper's emphasis on interpretability. The use of these features also makes it unclear how easy it would be to run the model in practice, which is also emphasized. I'd recommend also including an ablation study where these features are excluded.

5. Please report the missingness percentage per variable. Do the numbers in Supp Table 1 represent the imputed distribution?

Reviewer #3

(Remarks to the Author)
The article presents a study that leverages machine learning (ML) algorithms to predict the risk of invasive metastatic disease (IMD) in breast and lung cancer patients using a population-based dataset from Ontario, encompassing data from 143,341 patients collected between 2010 and 2023. The researchers developed two sets of models: one for IMD risk prediction and another for predicting IMD within five years of diagnosis. The ML models outperformed traditional statistical methods, demonstrating robust predictive capabilities, with key predictors such as histology, tumor site, age, and sex identified as significant factors for IMD risk.
While this research is indeed novel and intriguing, the data and code are not publicly available, which hampers the ability to validate the statistical analysis or reproduce the study's findings.
Further comments:
1. Please avoid using the term "globally" when the model is trained on data from a single location. Population diversity, mixed cases, and related factors could impact data composition, challenging the model's generalizability.
2. This research utilized only one model and one explanation method. In practical settings, ML models might tend to incorporate irrelevant information to enhance predictions. Employing multiple explanation mechanisms could reveal conflicting interpretations.
3.Please clarify whether there were any updates to diagnosis codes from 2010 to 2023 and how those updates might have impacted cohort selection.
4. Please explain why the train, validation, and test compositions were set at 49%, 21%, and 30%, respectively. Normally, the training set constitutes 70-80% of the data, with the remaining 10% for validation and 10-20% reserved for testing.

Version 1:

Reviewer comments:

Reviewer #1

(Remarks to the Author)
We appreciate the authors' responsiveness to the initial round of comments and substantial revisions to the manuscript, especially the change to a time-to-event analysis.

Major comments:
1. The use of an age-and-stage only comparator in Figure 3 is very helpful, as it best approximates current clinical practice. However, it is somewhat confusing to go from that comparator to figure 4, which uses a scan-all and scan-none as the two comparators (dashed lines), both of which are not current standard accepted clinical practice. Consider adding the age-and-stage comparator to figure 4 as well.

2. IPCW is referenced (lines 397 and 402) but the model used to calculate censoring weights is not described, nor how the censoring weights were balanced. Please explain how this was implemented.

3. The missingness table (S12) appears to not contain all relevant variables. For example, tumor size is listed as a significant predictor of lung cancer IMD (line 259) but is not listed in S12. Please include all variables.

Minor:
1. Please include a list of histology codes (small cell, adenocarcinoma, etc) or a reference to how they were defined for the different cancers.

2. Figure 1B - describe what the cumulative incidence is of (IMD?)

3. Figure 2 - describe A) and B) in the caption (cancer type?)

Reviewer #2

(Remarks to the Author)
I thank the authors for their updates. I have mostly minor comments remaining:

1. In addition to the stage/age baseline, I'd suggest including a standard (linear) Cox baseline that includes the same features included in the ML model. This will help determine whether the improved performance is due to the ML approach per se or the inclusion of multiple features. This baseline will also directly provide interpretable weights/HRs for each feature which can be compared to the ML interpretability analysis.

2. "Full baseline results are provided in Supplementary Table S4." - The C-index results do not appear to be included.

3. The description of the metholodogy behind the decision curve analysis is sparse. The parameters used and their justification are unclear.

4. In this sentence, "Differences over 0–5 years were significant by Gray's test ($p<0.001$), with absolute separations at 1, 3, and 5 years of 6.0, 8.4, and 8.4 percentage points for IMD and 39.9, 50.5, and 51.8 percentage points for death without IMD, respectively." It is unclear what the differences are referring to (i.e. differences between what)

Reviewer #3

(Remarks to the Author)
The authors have made appropriate and sufficient changes to the manuscript, and they have addressed my previous comments thoroughly.

Version 2:

Reviewer comments:

Reviewer #1

(Remarks to the Author)
The authors have commendably and rigorously addressed the requested revisions. There are no new revisions requested.

Reviewer #2

(Remarks to the Author)
I thank the authors the revisions. I have no additional comments.

Open Access This Peer Review File is licensed under a Creative Commons Attribution 4.0 International License, which

permits use, sharing, adaptation, distribution and reproduction in any medium or format, as long as you give appropriate credit to the original author(s) and the source, provide a link to the Creative Commons license, and indicate if changes were made.
In cases where reviewers are anonymous, credit should be given to 'Anonymous Referee' and the source.
The images or other third party material in this Peer Review File are included in the article’s Creative Commons license, unless indicated otherwise in a credit line to the material. If material is not included in the article’s Creative Commons license and your intended use is not permitted by statutory regulation or exceeds the permitted use, you will need to obtain permission directly from the copyright holder.
To view a copy of this license, visit https://creativecommons.org/licenses/by/4.0/

November 10, 2025

Response to Editor Comments

Manuscript Title: Machine learning identifies prognosticators of intracranial metastatic disease in patients with breast or lung cancer
Manuscript ID: NCOMMS-25-20713
Journal: Nature Communications
Corresponding Author: Sunit Das, MD, PhD (sunit.das@utoronto.ca)

We sincerely thank you for the time and effort you dedicated to reviewing our manuscript, and for your valuable and constructive feedback. We have carefully considered each comment and have revised the manuscript accordingly. Below, we provide a point-by-point response to all comments. Reviewer comments are reproduced in italics, followed by our responses in regular text.

Reviewer 1

Comment 1: The outcomes for the classification model are not clearly described. Specifically, it is unclear what proportion of the cohort died before they could develop a brain metastasis, and whether or how the competing risk of death was accounted for. Please describe.

We agree and have reframed the analysis explicitly as a competing-risks survival problem with two mutually exclusive endpoints: 1) intracranial metastatic disease and 2) death without prior IMD. In the breast cancer cohort (n=86,082), 2,665 (3.1%) developed IMD, 15,865 (18.4%) died without prior IMD, and 67,552 (78.5%) were censored without IMD or death. In the lung cancer cohort (n=57,259), 6,426 (11.2%) developed IMD, 39,065 (68.2%) died without prior IMD, and 11,768 (20.6%) were censored.

For prediction, our new analysis paradigm now fits cause-specific machine-learning Cox models for IMD and for death while patient-level IMD risk is obtained by combining the fitted cause-specific hazards via the Aalen–Johansen estimator to yield cumulative incidence functions that correctly account for the competing risk of death. Such models are now evaluated using time-to-event metrics, including the concordance index and time-dependent predictive statistics. This new paradigm encompasses the entirety of the *Results* section with concomitant reworking of the *Methods* and *Discussion*.

Comment 2: Please include a table of the rate of missingness in predictor variables in both cohorts.

Thank you for the suggestion. We have added a missing-data summary to the Supplement (see Supplementary Table S12), which reports for every predictor the number (N) and proportion (%) of missing values separately for the breast and lung cohorts. Predictors with no missingness are reported as 0%.

Comment 3: Please include a specific definition of time zero (presumably date of initial diagnosis?) and how the outcome of brain metastasis was defined.

We have clarified both concepts in the *Methods* (*Study Population and Variables*). Time zero is defined as the date of primary cancer diagnosis. The IMD outcome is defined as the first recorded diagnosis of intracranial metastatic disease identified across linked provincial datasets (Cancer Activity Level Reporting, Discharge Abstract Database, and National Ambulatory Care

Reporting System) using a prespecified ICD-10-CA code list as per the Institute of Clinical and Evaluative Sciences. The first coded instance in any source determined the IMD date. To avoid misclassifying pre-existing disease, we excluded synchronous IMD (≤7 days from cancer diagnosis).

Comment 4: Systemic treatment, specifically use of intra-cranially penetrant vs not, can significantly influence the development of brain metastases. Use has also changed significantly across the time period in the study (2010 - 2023). For example, the intracranially penetrant EGFR TKI osimertinib was introduced in 2015. It is unclear whether a model trained on data from a past era will continue to hold validity as systemic therapies change. Please justify a) using a validation test set that is randomly sampled across all time periods, instead of one split by time and b) why initial choice of systemic treatments are excluded from the model; or include as a limitation.

A. Random V Time-Based Split: Our primary objective was accurate baseline risk estimation for IMD under competing risks at fixed horizons. A strict time-split (train on early years, test on later years) would severely truncate follow-up for patients diagnosed late in the accrual window, making 3–5-year endpoints unattainable (e.g., diagnoses after mid-2018 cannot contribute complete 5-year outcomes by August 30, 2023). This inflates right-censoring, depresses event counts, and destabilizes horizon-specific metrics. By contrast, a random split sampled across all calendar years preserves effective sample size and event yield at each horizon, enabling more robust analysis. We now state this limitations and acknowledge that future temporal validation, training on earlier years and testing on later years with horizons feasible for later diagnoses, is desirable, along with prospective calibration monitoring to detect drift as therapies continue to change.

B. Exclusion of Initial System Treatment: Our intent is a baseline-only tool usable at diagnosis, before treatment decisions are finalized. Including initial systemic therapy would introduce post-baseline information into baseline prediction and risk immortal-time bias due to exposure to treatments not yet available at each evolving period (as described above). Ultimately, this would limit applicability at the point of decision-making when treatment is not yet known. We have clarified this design choice and now explicitly list the inclusions of systemic therapy as a future direction.

Comment 5: Small cell lung cancer histology is known to be significantly associated with increased risk of brain metastasis (https://www.jtocrr.org/article/S2666-3643(22)00054-6/fulltext). Justify why it is grouped under "Other" histology or separate the histology out.

Prior to modeling, we applied a uniform rarity-collapsing rule across all categorical predictors to mitigate sparse strata and unstable estimation in the competing-risks setting: across all categorical predictors, any level with <5% prevalence in the development set was collapsed into an “Other” category. SCLC had a prevalence of ~4.8% (with a 15% rate of IMD overall vs baseline of 11%) and was therefore amalgamated. This technical decision was taken as sparsity can cause instability of long-horizon IPCW-based discrimination/calibration and AJ-CIF estimates when a rare stratum is modeled separately. However, we acknowledge that this decision may attenuate histology-specific effects and have included it as a limitation.

November 10, 2025

***Comment 6: Please include a table of the specific " 34 comorbidity-based features" mentioned in line 311 of the methods*.**

Thank you for this suggestion. In recasting the analysis as a time-to-event problem and revising the manuscript, we found that retaining only the combined Aggregated Diagnosis Groups (ADGs) Score improved model parsimony and calibration without a material loss of discrimination. This score is based on work carried out at ICES (https://pubmed.ncbi.nlm.nih.gov/21921849/) and is cited in our paper. Accordingly, the final models use the combined ADG summary.

However, for reader accessibility, we have recapitulated the individual ADG indicators and the derivation of the ADG Score from the above paper into Supplementary Table S9.

Reviewer 2

Comment 1: The studied problem - risk of developing IMD over time - is best formulated as a time-to-event problem, where each patient is ultimately a) censored (according to latest follow up), b) experiences a competing risk (death), or c) experiences IMD. However, all the analysis is performed as a binary task (IMD event or not) and I can't find any discussion or reporting of follow-up times. This introduces risk of bias as patients may be assigned an outcome of "0" just because they had short follow up times. Even if the model is developed in the purely binary setting, it is important to evaluate it using time-to-event metrics such as the concordance index.

Thank you for this comment. This point overlaps with Reviewer #1, Comment #1 and a detailed response is provided there. In brief, we recast the analysis from a binary classification task to a time-to-event framework with a competing risk of death. We now (i) define mutually exclusive endpoints, IMD and death without prior IMD, (ii) report follow-up using reverse Kaplan–Meier and cohort-level Aalen–Johansen cumulative incidence of IMD at 1, 3, and 5 years, (iii) fit two cause-specific Cox models (IMD; death) and derive patient-level IMD risk via the Aalen–Johansen estimators, and (iv) evaluate performance with time-to-event metrics. This new paradigm encompasses the entirety of the *Results* section with concomitant reworking of the *Methods* and *Discussion*.

Comment 2: The ML model performance is currently lacking baselines. For instance, what is the predictive performance of stage alone? Table 1 reports a chi-squared p-value for univariates, but the actual predictive performance is not reported, making it unclear how much better the ML model is than baselines.

Thank you for this helpful suggestion. We agree that clear baselines are essential to contextualize performance. In the revision, we replaced the prior univariate χ^2 screening with cause-specific Cox effect sizes (HRs, 95% CIs) and added a stage-and-age-only baseline model evaluated identically to the ML models under competing risks. For all models, patient-level IMD risk is derived from cause-specific hazards via the Aalen–Johansen estimator, and performance is reported at 1, 3, and 5 years using Uno's C-index, time-dependent AUC/PR, integrated Brier score, calibration and decision-curve analysis.

Comment 3: The training/validation/test splitting is currently unclear. The methods first state that "The dataset was first split into training (49%), validation (21%), and test (30%) sets". Later, it is stated "To ensure robust model evaluation and mitigate overfitting, a five-fold

cross-validation (CV) strategy was adopted", and "Following model training on the validation set within each CV fold...". Separately, is there a reason for the non-round numbers of 49% and 21%?

Thank you for pointing this ambiguity out. We clarified the *Methods* to make the data partitioning unambiguous. Briefly, we use a two-stage scheme: a single, stratified 30% hold-out test set is sampled once from the full stratified cohort to preserve case-mix and event counts while the remaining 70% development set is used for model selection via 5-fold cross-validation (CV). Within CV, hyperparameters are tuned on the four training folds and evaluated on the one validation fold; folds are stratified identically. After tuning, the model is refit on the entire 70% development set and evaluated once on the untouched 30% test set with bootstrap CIs. The earlier '49%/21%/30%' wording reflected these equivalent proportions relative to the full cohort: within the 70% development set we targeted a 70/30 split across CV train/validation at any given fold (i.e., 0.7×0.7 ≈ 49% [train] and 0.7×0.3 ≈ 21% [validation] plus the fixed 30% test hold-out. We have removed this ambiguous language under *Methods, Date Preprocessing.*

Comment 4: The main text describes the clinical features included in the model such as stage, histology, etc. However, in the methods, it is also stated that 34 ADG features are also included in the model, which are not described in the main text besides brief mention in a figure caption. Given the large number of features, it's important to describe these in the main text and what they mean, especially given the paper's emphasis on interpretability. The use of these features also makes it unclear how easy it would be to run the model in practice, which is also emphasized. I'd recommend also including an ablation study where these features are excluded.

Thank you for this comment. This overlaps with Reviewer #1, Comment #6 and a complementary response is provided there. In brief, we clarified the comorbidity construct in the main text and simplified the feature set for practicality and interpretability. Specifically, we found that retaining only the combined Aggregated Diagnosis Groups (ADGs) Score improved model parsimony and calibration without a material loss of discrimination. This score is derived from previous work by ICES (https://pubmed.ncbi.nlm.nih.gov/21921849/). However, for transparency, we now enumerate all 34 ADGs with brief definitions and the mapping to the combined score in Supplementary Table S9.

Comment 5: Please report the missingness percentage per variable. Do the numbers in Supp Table 1 represent the imputed distribution?

Thank you for this comment. This overlaps with Reviewer #1, Comment #2 and a complementary response is provided there. We have added a missing-data summary to the Supplement (see Supplementary Table S12) which reports the requested rates of missingness. We have also clarified Supplementary Tables S10 and S11 to mention that it represents the pre-imputed distribution.

Reviewer 3

Comment 1: Please avoid using the term "globally" when the model is trained on data from a single location. Population diversity, mixed cases, and related factors could impact data composition, challenging the model's generalizability.

Thank you for this suggestion. With the new paradigm for evaluation, we develop time-to-event models that do not need to be named according to time. Consequently, we have avoided using the term globally.

Comment 2: This research utilized only one model and one explanation method. In practical settings, ML models might tend to incorporate irrelevant information to enhance predictions. Employing multiple explanation mechanisms could reveal conflicting interpretations.

Thank you for raising this. In the revision, we interrogate the model with two complementary explanation frameworks and report their agreement. Specifically, for each cause-specific model we compute Shapley Additive Explanations (SHAP) to capture local and global contributions and permutation importance on out-of-fold predictions and on the held-out test set to assess predictive reliance. We then form a rank-consensus across folds and explainers and report overlap of the top-ranked features. The rank-consensus results are reported in Figure 6 while the individual ranking for all predictors is reported in Supplementary Tables S5-S8.

Comment 3: Please clarify whether there were any updates to diagnosis codes from 2010 to 2023 and how those updates might have impacted cohort selection.

Ontario hospital and ambulatory encounters have been coded using ICD-10-CA/CCI continuously since 2002–2003, so our 2010–2023 study window does not span any transition from ICD-9 to ICD-10. During this window, CIHI released periodic version updates to ICD-10-CA and the Canadian Coding Standards (notably v2018 and v2022), but these did not alter the diagnosis code families underpinning our case definitions. Primary cancer site and histology come from the provincial cancer registry, which uses ICD-O-3 morphology/topography throughout; interim updates (ICD-O-3.2) were terminology refinements rather than structural changes that would affect our inclusion rules. We have included this exposition in *Methods, Study Population and Variables*.

Comment 4: Please explain why the train, validation, and test compositions were set at 49%, 21%, and 30%, respectively. Normally, the training set constitutes 70-80% of the data, with the remaining 10% for validation and 10-20% reserved for testing.

Thank you for this comment. This overlaps with Reviewer #2, Comment #3 and we present a complementary response here alongside a clarified Methods. In brief, we used a two-stage split. First, a single, stratified 30% hold-out test set was sampled. The remaining 70% development set was used for model selection via 5-fold cross-validation (CV), where hyperparameters were tuned on four training folds and evaluated on one validation fold. The final model was then refit on the entire 70% development set and evaluated once on the untouched 30% test set.

We removed previous, confusing "49%/21%/30%" language (which reflected notional proportions) and now explicitly state that validation folds are only for tuning and the test set is never used for training or selection. This approach supports our competing-risks evaluation and avoids follow-up truncation.

February 9, 2026

Response to Editor and Review Comments

Manuscript Title: Machine learning identifies prognosticators of intracranial metastatic disease in patients with breast or lung cancer
Manuscript ID: COMMSMED-25-0581B
Journal: Nature Communications
Corresponding Author: Sunit Das, MD, PhD (sunit.das@utoronto.ca)

We sincerely thank you for the time and effort you dedicated to reviewing our manuscript, and for your valuable and constructive feedback. We have carefully considered each comment and have revised the manuscript accordingly. Below, we provide a point-by-point response to all comments. Reviewer comments are reproduced in blue italics, followed by our responses in regular text.

Reviewer 1

Comment 1: The use of an age-and-stage only comparator in Figure 3 is very helpful, as it best approximates current clinical practice. However, it is somewhat confusing to go from that comparator to figure 4, which uses a scan-all and scan-none as the two comparators (dashed lines), both of which are not current standard accepted clinical practice. Consider adding the age-and-stage comparator to figure 4 as well.

Thank you for this suggestion. We agree that Figure 4 should be anchored to clinically relevant comparators rather than only the treat-all and treat-none reference strategies. In response (and under advice of Reviewer #2 Comment #1), we expanded our comparator set to include (i) an age-and-stage-only baseline and (ii) a linear Cox model trained on the full covariate set (i.e., the same covariates as the complete tree-ensemble model), enabling us to disentangle gains from additional covariates versus gains from nonlinear modeling.

In the updated analyses, the linear Cox (all covariates) model was the strongest-performing comparator; therefore, to avoid overcomplicating the main figure while still providing a clinically meaningful benchmark, we present Figure 4 as a comparison between the full tree-ensemble AJ-CIF model and the best-performing comparator (linear Cox; all covariates), with treat-all and treat-none retained as decision-curve references. We have updated Figure 4 and *Results: Comparison with Baseline Models* accordingly. Full decision-curve results for all evaluated comparators, including the age-and-stage baseline, are provided in Supplementary Table S5 so readers can directly assess clinical utility relative to both comparators.

If preferred by the editors, we can additionally include the age-and-stage curve in the main Figure 4; however, we opted to prioritize readability and show the strongest comparator in the main text while providing the full set in the Supplement.

Comment 2: IPCW is referenced (lines 397 and 402) but the model used to calculate censoring weights is not described, nor how the censoring weights were balanced. Please explain how this was implemented.

Thank you for flagging this. We now detail the IPCW implementation in *Methods: Model Evaluation*. Briefly, all time-dependent metrics use the cumulative/dynamic definition: at horizon t, cases are patients who developed IMD by t, controls are event-free at t, and deaths before t are treated as competing events and excluded beyond their death time. The censoring survival, ,

$\hat{G}(t)$, is estimated non-parametrically with a Kaplan–Meier estimator on the evaluation split only (the validation fold during cross-validation or the held-out test set for final evaluation) to avoid leakage and reflect split-specific censoring. IPCW weights are then formed as $w_i(T_i) = \frac{1}{\hat{G}(T_i)}$ for cases at their event time and $w_i(\mathrm{t}) = \frac{1}{\hat{G}(\mathrm{t})}$ for controls at horizon t. To stabilize estimation, weights are truncated at the 1st and 99th percentiles and renormalized at each evaluation time so the mean weight is approximately 1. All weighting (KM estimation and derived IPCW) is recomputed within each CV validation fold and on the held-out test set; for test-set bootstrap CIs, weights are recomputed within each resample.

Comment 3: The missingness table (S12) appears to not contain all relevant variables. For example, tumor size is listed as a significant predictor of lung cancer IMD (line 259) but is not listed in S12. Please include all variables.

Thank you for catching this oversight. We have expanded Supplementary Table S12 (S13 in this revision) to include all variables used in modelling and reporting of the final models, not just those used for the calculation of the Cause-Specific Hazard Models (Figure 2). Specifically, we added tumor size, tumor grade, and laterality. Conventions remain the same, with missingness reported pre-imputation and separately by cancer cohort (breast, lung) and dataset (development, validation).

Comment 4: Please include a list of histology codes (small cell, adenocarcinoma, etc) or a reference to how they were defined for the different cancers.

Thank you for this request. We have clarified histology definitions in *Methods: Study Population and Variables*. Histology was derived from ICD-O-3 morphology–based codes using a prespecified ICES list applied uniformly from Jan 1, 2010 to Aug 30, 2023; the 2013 and 2019 revisions did not alter these definitions. Because histology cardinality is high and to avoid sparse strata, categories with <5% prevalence in the development set were collapsed into *Other* (as stated in Methods). We have updated Supplementary Tables S9–S10 to list the ICD-O code sets for all retained (≥5%) histology categories alongside plain-language labels.

Per ICES small-cell disclosure policy, granular code lists for small-cell and other potentially identifying rare categories cannot be publicly released. Accordingly, these appear within the 'Other' category in public materials; however, their membership is defined from the same prespecified ICD-O code list and was applied uniformly across years. Researchers can reproduce the full mapping within the ICES environment under a data-sharing agreement; access details are provided in the *Data Availability* statement.

Comment 5: Figure 1B - describe what the cumulative incidence is of (IMD?).

Thank you for the clarification request. We have updated Figure 1B and its caption to state explicitly that the curve shows the Aalen–Johansen cumulative incidence of IMD under the competing risk of death.

Comment 6: Figure 2 - describe A) and B) in the caption (cancer type?)

Thank you for the clarification request. We now explicitly label the panels by cancer type and summarize what is plotted.

February 9, 2026

Reviewer 2

Comment 1: In addition to the stage/age baseline, I'd suggest including a standard (linear) Cox baseline that includes the same features included in the ML model. This will help determine whether the improved performance is due to the ML approach per se or the inclusion of multiple features. This baseline will also directly provide interpretable weights/HRs for each feature which can be compared to the ML interpretability analysis.

Thank you for your feedback. We have added a linear Cox comparator trained on the same full covariate set used by the tree-ensemble model to distinguish gains due to inclusion of additional covariates from gains due to nonlinear modeling. Specifically, we trained cause-specific linear Cox models for IMD and for death using the identical pipeline (same preprocessing, splitting, and evaluation framework), and combined the fitted cause-specific hazards via the Aalen–Johansen estimator to generate AJ-CIF absolute-risk predictions. We report this comparator throughout the revised manuscript in both the *Results: Comparison with Baseline Models* and *Methods: Model Evaluation* alongside the original minimal age-and-stage-only comparator.

In the updated discrimination analyses, the linear Cox (all covariates) models achieved Uno's C-index of 0.83 for breast IMD (CV: 0.83 ± 0.01) and 0.75 for lung IMD (CV: 0.75 ± 0.01), compared with 0.79 and 0.74 for the age-and-stage-only baselines, respectively. While inclusion of additional covariates improved performance relative to age/stage alone, the full tree-ensemble models remained superior, with Uno's C-index for IMD 15% higher than linear Cox in breast cancer and 17% higher in lung cancer (death: 2% and 7% higher, respectively). Full model performance comparisons are provided in Supplementary Table S4, and we updated Supplementary Figures S4a/S4b to include these models and metrics.

For clinical utility, we simplified the main-text decision-curve figure to compare the full tree-ensemble AJ-CIF models against the best-performing baseline (linear Cox; all covariates) and updated the decision-curve analyses accordingly (Figure 4; Supplementary Table S5).

Comment 2: "Full baseline results are provided in Supplementary Table S4." - The C-index results do not appear to be included.

We thank the reviewer for noting this. Supplementary Table S4 reports performance metrics for the AJ-CIF absolute-risk models, whereas Uno's C-index is computed for the underlying cause-specific Cox models (pre–AJ-CIF) and therefore was not shown in that table. Recognizing that we did not report the absolute comparator C-index values in the original manuscript, we have corrected this in the revision by explicitly providing Uno's C-index for both the developed models and all comparator models, along with the cross-validated mean and standard deviation. The developed-model C-indices are reported in *Results: Survival Machine Learning Models: Discrimination of the Cause-Specific Models*, and the comparator-model C-indices are reported in *Results: Comparison with Baseline Models*.

Comment 3: The description of the methodology behind the decision curve analysis is sparse. The parameters used and their justification are unclear.

We thank the reviewer for this comment and agree that additional methodological detail was needed. We have revised *Methods: Model Evaluation* to provide a clearer, more explicit description of our time-dependent decision-curve analysis, including the inputs, parameters, and justification for the chosen threshold range. Specifically, we now state that decision-curve analysis was performed at 1, 3, and 5 years using each model's predicted AJ-CIF IMD risk at the

corresponding horizon; thresholds were evaluated from 0% to 10% in 0.5 percentage-point increments, with patients above a threshold classified as “test-positive.” We clarify comparison against treat-none and treat-all reference strategies, report both net benefit and net reduction in unnecessary interventions (per 100 patients) and describe how right-censoring was handled via IPCW with death treated as a competing event, consistent with our time-dependent performance metrics. Finally, we added an explicit a priori justification for focusing on the 0–10% threshold range as clinically plausible action thresholds for IMD surveillance.

Comment 4: In this sentence, "Differences over 0–5 years were significant by Gray’s test (p<0.001), with absolute separations at 1, 3, and 5 years of 6.0, 8.4, and 8.4 percentage points for IMD and 39.9, 50.5, and 51.8 percentage points for death without IMD, respectively." It is unclear what the differences are referring to (i.e. differences between what).

We thank the reviewer for highlighting this ambiguity. We have revised the caption of Figure 1 to explicitly state the comparison being made (breast versus lung cancer) and clarified that both the IMD CIF and the death-without-prior-IMD CIF differed significantly between the two cohorts over 0–5 years (Gray’s test, p<0.001).
